# Neisserial adhesin A (NadA) binds human Siglec-5 and Siglec-14 with high affinity and promotes bacterial adhesion/invasion

**DOI:** 10.1128/mbio.01107-24

**Published:** 2024-07-23

**Authors:** Barbara Benucci, Zaira Spinello, Valeria Calvaresi, Viola Viviani, Andrea Perrotta, Agnese Faleri, Sabrina Utrio Lanfaloni, Werner Pansegrau, Liana d’Alterio, Erika Bartolini, Irene Pinzuti, Katia Sampieri, Anna Giordano, Rino Rappuoli, Mariagrazia Pizza, Vega Masignani, Nathalie Norais, Domenico Maione, Marcello Merola

**Affiliations:** 1 GSK, Siena, Italy; 2 Department of Pharmacy, University of Copenhagen, Copenhagen, Denmark; 3 Università di Siena, Siena, Italy; 4 Università di Napoli Federico II, Naples, Italy; College of Veterinary Medicine, Cornell University, Ithaca, New York, USA

**Keywords:** Neisserial adhesin A (NadA), NadA receptor, *Neisseria meningitidis*, Siglec-5, Siglec-14, Siglec-9

## Abstract

Neisserial adhesin A (NadA) is a meningococcal surface protein included as recombinant antigen in 4CMenB, a protein-based vaccine able to induce protective immune responses against *Neisseria meningitidis* serogroup B (MenB). Although NadA is involved in the adhesion/invasion of epithelial cells and human myeloid cells, its function in meningococcal physiology is still poorly understood. To clarify the role played by NadA in the host-pathogen interaction, we sought to identify its cellular receptors. We screened a protein microarray encompassing 2,846 human and 297 mouse surface/secreted recombinant proteins using recombinant NadA as probe. Efficient NadA binding was revealed on the paired sialic acid-binding immunoglobulin-type lectins receptors 5 and 14 (Siglec-5 and Siglec-14), but not on Siglec-9 therein used as control. The interaction was confirmed by biochemical tools with the determination of the *K*_*D*_ value in the order of nanomolar and the identification of the NadA binding site by hydrogen-deuterium exchange coupled to mass spectrometry. The N-terminal domain of the Siglec-5 that recognizes the sialic acid was identified as the NadA binding domain. Intriguingly, exogenously added recombinant soluble Siglecs, including Siglec-9, were found to decorate *N. meningitidis* surface in a NadA-dependent manner. However, Siglec-5 and Siglec-14 transiently expressed in CHO-K1 cells endorsed NadA binding and increased *N. meningitidis* adhesion/invasion while Siglec-9 did not. Taken together, Siglec-5 and Siglec-14 satisfy all features of NadA receptors suggesting a possible role of NadA in the acute meningococcal infection.

**IMPORTANCE:**

Bacteria have developed several strategies for cell colonization and immune evasion. Knowledge of the host and pathogen factors involved in these mechanisms is crucial to build efficacious countermoves. Neisserial adhesin A (NadA) is a meningococcal surface protein included in the anti-meningococcus B vaccine 4CMenB, which mediates adhesion to and invasion of epithelial cells. Although NadA has been shown to bind to other cell types, like myeloid and endothelial cells, it still remains orphan of a defined host receptor. We have identified two strong NadA interactors, Siglec-5 and Siglec-14, which are mainly expressed on myeloid cells. This showcases that NadA is an additional and key player among the *Neisseria meningitidis* factors targeting immune cells. We thus provide novel insights on the strategies exploited by *N. meningitidis* during the infection process, which can progress to a severe illness and death.

## INTRODUCTION

Neisserial adhesin A (NadA) is a meningococcal membrane protein identified by reverse vaccinology (1, 2) and included as recombinant soluble antigen in the anti-meningococcus B vaccine (4CMenB) along with factor H binding protein (fHbp), neisserial heparin binding antigen (NHBA), and outer membrane vesicles from the New Zealand strain (3). NadA is highly immunogenic, induces protective bactericidal responses, and has self-adjuvating activity (4).

The *nadA* gene is present in ~30% of *Neisseria meningitidis* major disease-associated strains and is mostly associated with three out of four hypervirulent *N. meningitidis* serogroup B lineages (5, 6). The NadA protein is classified into two genetically and immunologically distinct groups: group I comprises variants 1, 2, and 3 (vaccine variant), while group II includes the rarer variants 4, 5, and 6 (5). NadA variant 3, used in this study, is the vaccine variant and hereinafter will be referred as NadA. Expression levels of the adhesin vary among isolates by more than 100-fold, and they are induced by appropriate niche-specific signals during colonization and infection (7).

A role of NadA in the adhesion process was inferred via its sequence similarity with UspA2 and YadA of *Moraxella catarrhalis* and *Yersinia enterocolitica,* respectively (8). NadA, belonging to the trimeric autotransporters (TAAs) family of adhesins, shows a tripartite organization comprising a β-barrel transmembrane anchor (anchor; amino acids 351–405), a long central region organized in α-helices (stalk; amino acids 90–350 approximately), and a more variable N-terminal region (head; amino acids 24–89 approximately), which confers specificity to the binding (9, 10). The stalk and head domains are projected toward the extracellular milieu and are together referred to as passenger domain. The X-ray structures of NadA variants 3 and 5 have revealed a unique and distinct folding of the N-terminal head region with no close homologs (10, 11). The bulky head domain results from an opening of the coiled-coil, forming a homo-tripartite wing-like structure laying lateral of an N-terminal coiled-coil segment. Contrary to other TAAs, the passenger domain of NadA spontaneously folds into a stable trimeric structure that allows its expression and purification as recombinant soluble antigen without the anchor domain (12, 13).

Early reports have shown that human epithelial cells were specifically targeted by NadA (8, 14). However, it was later observed that recombinant NadA was also able to bind human monocytes, thereby inhibiting apoptosis and inducing differentiation into macrophages (15). Furthermore, NadA binding to macrophages and monocytes-derived dendritic cells resulted in secretion of proinflammatory cytokines (15, 16). In both human monocytes and epithelial cell types, external heat shock protein 90 (Hsp90) was found to be involved in directing the biological signaling driven by NadA (13, 17, 18).

The specific cellular receptors able to bind NadA are still poorly understood. Among them, β1-integrin has been indicated as a potential epithelial NadA cell receptor (19). Moreover, NadA has been shown to have a high binding affinity to the scavenger lectin-type oxidized LDL receptor-1 (Lox-1), which is mainly expressed on endothelial cells (20). Intriguingly, the binding of NadA to this specific cell type has been reported only recently (21). Although the biological relevance of these findings has not been elucidated yet, it is remarkable that NadA could potentially have a role on different cell types encountered by the pathogen during the progression of the invasive meningococcal disease (22).

Sialic acid-binding immunoglobulin-type lectins (Siglecs) family comprises 14 members in human, with individual Siglec showing cell type specific patterns of expression (23, 24). The extracellular domains of these type I transmembrane proteins comprise the ligand-binding V-set Ig domains (consisting of the first two N-terminal IgG domains), which act as receptors for sialic acids, and a variable number of C2-set Ig domains (23, 25). Siglecs are important regulators of homeostasis in both innate and adaptive immune responses and are the major family of sialic acid receptors. Indeed, sialic acids are present on all human cell types, and the binding of sialoglycans to these receptors is a physiological mechanism to control inflammation and immune activation against self-antigens (26). Likewise, bacteria and virus have evolved strategies to decorate themselves with sialic acids in order to subvert the immune response and to ensure progression of the disease or infection (27, 28). Most of the members of the Siglec family, including Siglec-5 and Siglec-9, mediate inhibitory cellular signaling via the immunoreceptor tyrosine-based inhibitory motif (ITIM). Although Siglec-14 derives from Siglec-5 gene duplication, it belongs to activating-type Siglecs, as it stimulates the immunoreceptor tyrosine-based activating motif (ITAM) on DAP12 associated molecules (29). Siglec-5 and Siglec-14 represent the first example of paired receptors owning the same substrate specificity but opposite cellular signaling (30). Moreover, their sialic acid binding sites, spanning the first two N-terminal Ig domains, are identical except for one amino acid (30). The evolution of paired Siglecs is believed to represent a host defense mechanism to counteract inhibitory signaling exploited by sialic acids carried by some pathogens (31–33).

Bacterial adhesion is a crucial step in host-pathogen interaction, and it is a common trait shared by commensal as well as pathogenic bacteria. For pathogens, a second phase including colonization and/or cellular invasion follows (34, 35). In this case, most often, microorganisms must fight local conditions of clearance, such as fluid shearing or mucosal secretion. Due to the importance of maintaining proximity to or being internalized into their host, they have developed several redundant functions to tightly adhere to host cells (36). The importance of non-pili adhesins has emerged as a significant peculiarity of bacterial adherence and the cooperativity of all these components is crucial to determine a successful colonization (34, 35). Identification of bacterial adhesion targets received increasing attention for its potential prophylactic and therapeutic applications, such as antigens in vaccines, targets for immunotherapy, soluble receptors, or adhesins analogs to block interaction (34, 35). In this report, we used a large-scale protein microarray made of 2,846 humans and 297 mouse individual recombinant proteins to identify NadA putative receptors. Using the recombinant soluble NadA (NadA_24–350_ hereinafter referred as rNadA) as a probe, we found novel NadA interactors, including the human paired receptors Siglec-5 and Siglec-14. The binding of NadA to these receptors was further validated by surface plasmon resonance (SPR) and hydrogen-deuterium exchange mass spectrometry (HDX-MS) experiments. We then investigated Siglec-NadA interaction both on eukaryotic and prokaryotic cells. Our data provide molecular evidence of the previously reported NadA-driven interaction of *Neisseria meningitidis* to human myeloid cells and suggest a pivotal role of NadA in the invasive phase of the meningococcal disease.

## RESULTS

### Protein microarray revealed the interaction between rNadA and Siglec-5 and Siglec-14

To identify potential NadA interactors/receptors, a protein microarray screening was performed using an elaborate microarray generated by spotting individual human and mouse recombinant soluble proteins, mostly spotted in more copies from different preparations, from an expanded GNF protein library (20, 37). The represented species were 2,846 and 297 of human and mouse origins, respectively (Table S1). One micromolar of rNadA was used as probe, and the specific binding was revealed using rabbit anti-NadA polyclonal antibodies and fluorescent anti-rabbit secondary antibodies. Signals 15,000 ≤ mean fluorescence intensity (MFI) < 30,000 and 5,000 ≤ MFI < 15,000 were revealed for medium and low reactive proteins, respectively (indicated in orange and yellow in Table S1) while proteins providing an MFI value inferior to 5,000 were considered non-reactive. Signals higher than 30,000 MFI were chosen as threshold for putative interactors and 19 highly reactive human hits fitted in this range (in bold in Table 1).

**TABLE 1:** Protein microarray screening of NadA interactors^*a*^^,*b*^

Protein name	Alias	Gene ID	Locus	MFI
Sialic acid binding Ig-like lectin 14	SIGLEC14	100049587	NP_001092082	**65,471**
				**65,477**
Protein tyrosine phosphatase receptor type S isoform 4	PTPRS	5802	NP_570925	**65,458**
Vasoactive intestinal peptide	VIP	7432	NP_003372	**65,446**
				**60,843**
				**55,475**
				169
Oxidized low density lipoprotein receptor 1	OLR1	4973	NP_002534	**65,405**
				**64,665**
				**64,284**
				**63,082**
				**61,674**
Stathmin 2	STMN2	11075	NP_001186143	**65,380**
				**63,254**
				**61,352**
Interleukin 6	IL6	3569	NP_000591	**65371**
				13,854
Protein tyrosine phosphatase receptor type S isoform 3	PTPRS	5802	NP_570923	**65,098**
Ghrelin and obestatin prepropeptide	GHRL	51738	NP_001128413	**64,641**
Megakaryocyte and platelet inhibitory receptor G6b	MPIG6B	80739	NP_079536	**61,064**
				**52,330**
				427
				360
				112
				75
Fc mu receptor	FCMR	9214	NP_001135945	**62,596**
				422
				251
Sperm acrosome associated 7	SPACA7	122258	NP_660291	**58,590**
				18,228
Motilin	MLN	4295	NP_001035198	**58,619**
				25,048
				3,288
				2,519
				111
Sialic acid binding Ig-like lectin 5	SIGLEC5	8778	NP_003821	**58,551**
Cancer/testis antigen 83	CT83	203413	NP_001017978	**58,338**
				2,185
Fc gamma receptor IIa	FCGR2A	2212	NP_001129691	**46,609**
				11,201
Interleukin 21	IL21	59067	NP_068575	**44,309**
				2,456
				798
Annexin A2	ANXA2	302	NP_001002857	**43,308**
Ribosome maturation factor	TSR3	115939	NP_001001410	**43,016**
				316
Chromosome two open reading frame 66	C2orf66	401027	NP_998773	**41,151**

^*a*^ Plotting of MFI values of spotted soluble recombinant human proteins tested against rNadA_24–350_ at 1 µM. Only highly reactive proteins (MFI ≥ 30,000) are reported, and all the other hits are listed in Table S1. Siglec-9 was among the non-reactive proteins and scored 47 MFI (Table S1).

^*b*^ Bold indicates values higher than 30,000 MFI.

Protein arrays are a powerful unbiased tool to identify potential interactors, but more refined analyses are required to validate a strong molecular binding. A first selection was done considering, among the high reactive proteins, coherent range of signal in all samples and excluding species whose duplicates ranged in different areas of reactivity. This group included CT83, FCGR2A, FCMR, IL6, IL21, MLN, MPIG6B, SPACA7, TSR3, and VIP. We adverse selected PTPRS since we found that it was highly reactive with all kinds of probes used in this and previous works (L. Scietti and B. Benucci, personal communication). As NadA is an adhesin that provides adhesion/invasion features to the organism that expresses it, we looked at potential cell receptors among the remaining proteins allowing exclusion of other candidates. STMN2 is mainly expressed in the brain and is involved in signal transmission likely anchored to the internal side of the plasma membrane, such as ANXA2 member of calcium-dependent phospholipid binding protein family. C2orf66 is a small uncharacterized protein expressed at low levels in a restricted number of tissues, while GHRL is a secreted hormone mainly expressed by stomach and duodenum. Finally, we did not consider positive hits from mouse proteins (Table S1). The three remaining positive hits showed receptor characteristics. Indeed, ORL1, mainly expressed on endothelial cells, was previously identified as NadA putative receptor (20). Two other proteins, belonging to the same family of sialic acid binding proteins, were positive to the screening: the paired receptors Siglec-14 and Siglec-5 (Table 1). These receptors are expressed principally on the human myeloid cells, a cell type already shown to bind rNada (15). Interestingly, these two proteins differ for only one amino acid in their sialic acid binding domain, suggesting a specific targeting of the adhesin. Other members of the family were present on the array, i.e., Siglec-3, Siglec-6, Siglec-7, Siglec-9, and Siglec-10, and they all resulted non-reactive toward NadA (Table S1). Thus, we decide to explore deeper the strength of the NadA interaction with Siglec-5/Siglec-14 using Siglec-9 as negative control in the ensuing experiments.

### Biochemical characterization of the binding speciﬁcity between rNadA and rh-Siglecs

**Fig 1:**
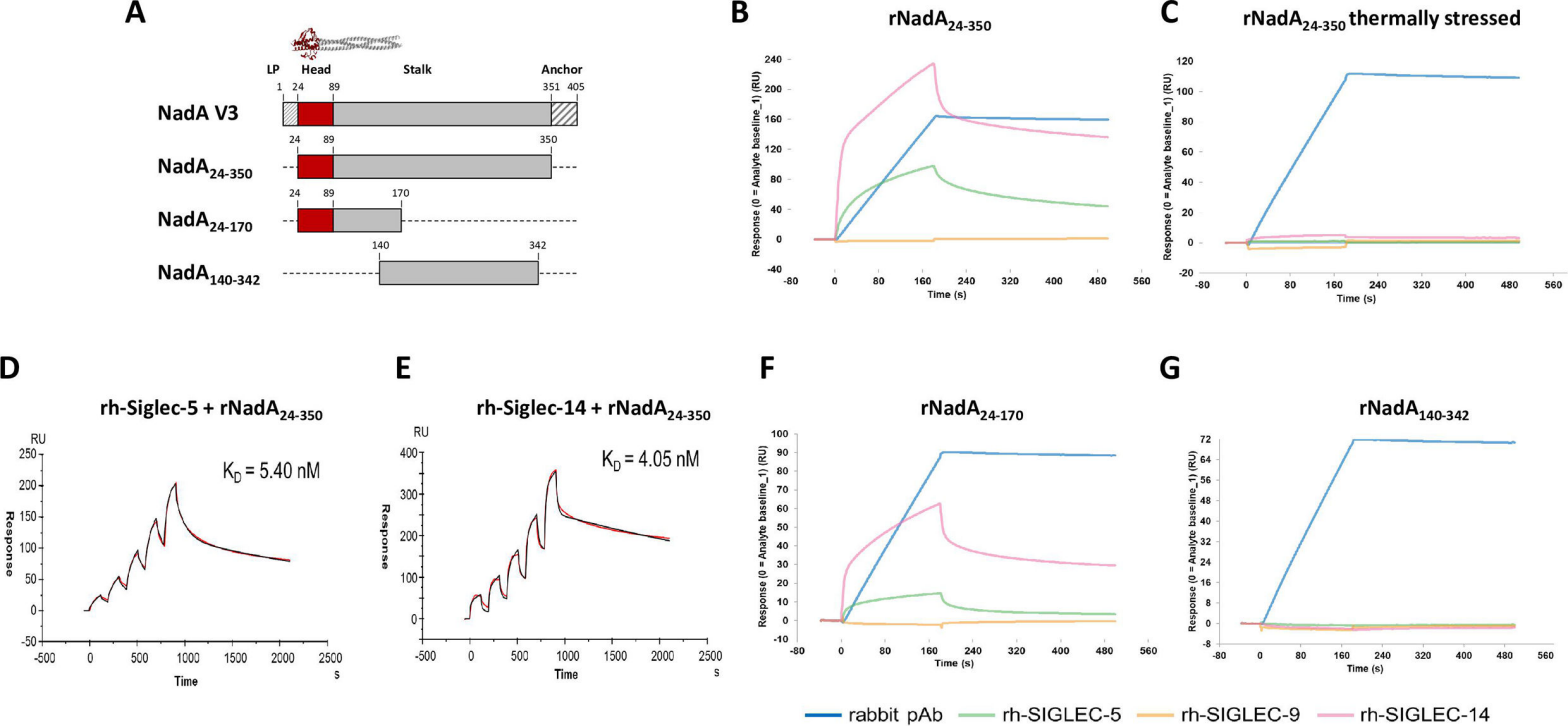
Surface plasmon resonance analyses of NadA-Siglec interactions. (**A**) Schematic representation of generated NadA fragments. NadA V3 represents the full-length molecule (crystal structure of NadA_24–170_; Protein Data Bank [PDB]: 6EUN); NAdA_24–350_ also referred as rNadA or NadA ectodomain lacks the leader peptide and the anchor domain; NadA_24–170_ represents the trimeric NadA head domain also containing part of the stalk, and NadA_140–342_ contains the stalk domain. Binding analysis between immobilized unstressed (**B**) and thermally stressed (**C**) rNadA and Siglec proteins (pink, rh-Siglec-14; orange, rh-Siglec-9; green, rh-Siglec-5). Single cycle kinetics of Siglec-5 (**D**) and Siglec-14 (**E**) proteins with immobilized rNadA as ligand, respectively. The graphs represent sensorgrams (red) and fitted curves (black) after injection of Siglecs at increasing concentrations. Curve fitting in both cases was performed using the two-state binding model. Binding analysis between NadA_24–170_ (**F**) or NadA_140–342_ (**G**) and Siglec proteins (pink, rh-Siglec-14; orange, rh-Siglec-9; green, rh-Siglec-5). In panels B, C, F, and G, rabbit anti-NadA polyclonal sera were used as positive controls (blue). For each sensorgram, blank subtraction was performed by subtracting the signal obtained with injection of buffer alone instead of Siglec proteins. Plot abscissa represent time in seconds, while ordinates represent the binding response measured in resonance units (RU).

**Fig 2:**
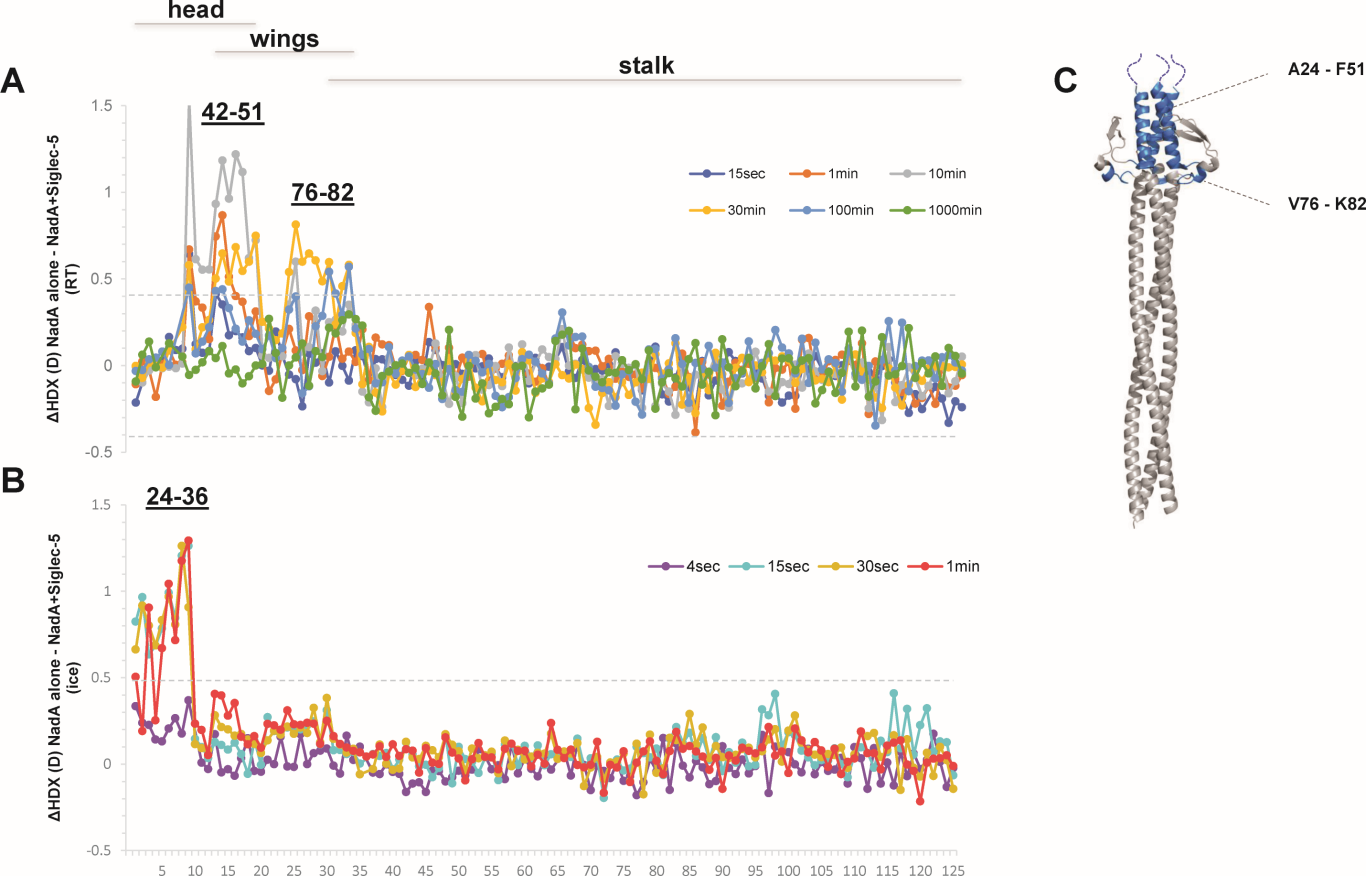
Conformational impact of rh-Siglec-5 on rNadA. Difference plots illustrate difference in HDX (*y*-axis) over the measured time points upon deuterium labelling at room temperature (**A**) and on ice (**B**) for the 125 peptides considered for the analysis (*x*-axis). Peptides are numbered along the *x*-axis according to their position from the N- to the C-terminus of the protein. Dotted lines are plotted at ±0.36D room temperature (RT) and ±0.40D (ice) indicating the 98% confidence interval (CI) as a threshold of significance for the effect. (**C**) Regions showing a difference in dynamics are colored on the crystal structure of NadA_24–170_ (PDB: 6EUN), added with dashed lines at the N-terminal sequence for which no structural determination is available.

Surface plasmon resonance (SPR) was used to investigate the binding kinetics between NadA and recombinant human Siglec-5 and Siglec-14 (rh-Siglec-5 and rh-Siglec-14, respectively) putative receptors (Fig. 1). Unstressed trimeric and thermally stressed monomeric NadA ectodomains (Fig. 1A) were firstly immobilized onto separate sensor chip flow cells and the binding was monitored by injecting a fixed concentration of rh-Siglecs. Rh-Siglec-9 and rabbit polyclonal anti-NadA antibodies were used as negative and positive controls, respectively. The obtained sensorgrams revealed strong rh-Siglec-5 and rh-Siglec-14 recognitions of folded NadA, while all the rh-Siglecs tested lacked any binding on the stressed NadA (Fig. 1B and C, respectively), suggesting a conformation-dependent binding of rh-Siglec-5 and rh-Siglec-14 toward NadA. To determine the *K*_*D*_ of NadA-Siglec associations, we performed kinetics at increasing concentration of the probes. As shown in Fig. 1D and E, a *K*_*D*_ in the nanomolar range was calculated for both lectin receptors: 5.40 and 4.05 nM for rh-Siglec-5 and rh-Siglec-14, respectively. To identify the specific domain of NadA involved in the binding, we tested the ability of two emimolecules (NadA_24–170_ and NadA_140–342_; Fig. 1F and G) to interact with the recombinant receptors. Considering that the binding of rh-Siglecs to NadA required a quaternary NadA structure, the NadA recombinant fragments were cloned and expressed as molecules with the minimum size but maintaining their folding. In particular, in the NadA_24–170_, half of the stalk coiled-coil structure was removed, but the N-terminal head domain resulted structured (10). Conversely, NadA_140–342_ lacked the head and wing-like structures but carried the stalk domain correctly trimerized (M. J. Bottomley, personal communication). As shown in Fig. 2F and G, both rh-Siglec-5 and rh-Siglec-14 were able to bind NadA_24–170_ yet did not bind the stalk domain (NadA_140–342_), which was exclusively recognized by the control anti-NadA sera. No binding was observed with Siglec-9 in any tested condition.

All together, these data attested that the soluble moiety of Siglec-5 and Siglec-14 establishes a high affinity binding to rNadA and specifically to the amino-terminal head domain of NadA.

### HDX-MS analysis of rNadA in complex with Siglec-5

To elucidate the conformational impact of the binding of Siglec-5 to rNadA, we carried out HDX-MS analysis on rNadA alone and in complex with the lectin (Tables S2 and S3) . We followed the deuterium incorporation of 125 peptides (average redundancy of 6.28), spanning the whole protein sequence. Spatially resolved information almost at single amino acid level were obtained for most of the protein sequence. By labeling at room temperature (RT), we observed a significant decreased HDX in the presence of Siglec-5 on the head domain of NadA. By comparing overlapping peptide, we could more specifically localize the binding effect in the region spanning residues Y42 to F51 and V76 to K82, corresponding to the third and fourth helices of the head domain and part of the connecting segments between head and wings (Fig. 2A; supplemental material uptake plots in Fig. S1). Intriguingly, we observed that peptides 24–35, 24–37, and 24–39, displayed very high deuterium incorporation already after 15 s of deuterium labeling (deuterium uptake plots in Fig. S1); therefore, they comprise a highly flexible region. We reasoned that this protein segment could potentially display differences in HDX in the sub-second time scale, remaining undetected within the kinetic window studied at RT. Therefore, in order to comprehensively investigate the conformational impact of Siglec-5 on the highly dynamic N-terminus of the head domain, the time window of our experiment was expanded by decreasing the exchange reaction temperature (deuterium labeling performed on ice) (38–41). The experiment performed on ice showed protection to HDX upon Siglec-5 binding also in the region spanning amino acids A24 to V36 (Fig. 2 and uptake plots in Fig. S2), encompassing the first and second helices of the head domain. No significant HDX changes were observed for peptides encompassing the stalk of the adhesin either at RT or on ice. To depict a complete map of the conformational impact of Siglec-5 on NadA structure, we examined together the difference in HDX elucidated under both ice and RT conditions. By mapping peptides showing variation in HDX under at least one of the two temperatures studied (Fig. S3), we identified HDX effects induced by the receptor on the whole coiled-coil head domain of the adhesin (residues A24–F51) and its connecting segments to the wings (residues V76–K82) (Fig. 2C).

### NadA binding to Siglec-5/Siglec-14 is barely detectable on PBMC-derived monocytes and neutrophils

**Fig 3:**
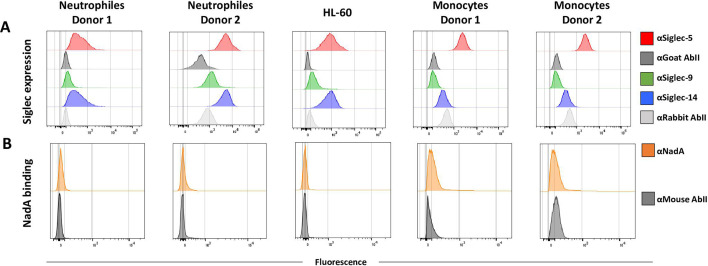
Siglec-5, Siglec-9, and Siglec-14 expressions and rNadA binding to human monocytes and neutrophils. Cells were incubated with human serum to block Fcγ receptors. Assay was performed in the presence of cytochalasin to avoid internalization. (A) The expression of Siglec-5, Siglec-9, and Siglec-14 on human monocytes and neutrophiles was assessed by cytofluorometry. The gray curves (αGoat AbII and αRabbit AbII) represent the secondary antibodies alone. Colored curves represent the surface exposure of different Siglecs. (B) rNadA binding on these same cells (orange profiles) was assessed by FACS using mouse 9F11 anti-NadA antibodies and compared with secondary antibody alone as control.

Siglec-5 and Siglec-14 are mainly expressed on myeloid cells (24). We sought to verify whether rNadA was also able to recognize the two interactors in cells that physiologically express these receptors. We performed NadA binding on monocytes and neutrophils isolated from human donors and on the neutrophils-like HL-60 cell line. As reported previously (13), rNadA binding to cells requires physiological temperature at least for 30 min and cannot be performed on ice as for antibodies. On eukaryotic cells, these conditions could activate an internalization pathway that would hamper the detection of the adhesin. Therefore, we used cells pretreated with cytochalasin, an inhibitor of actin polymerization, to block endocytosis. Firstly, we checked the presence of Siglec-5, Siglec-14, and the control Siglec-9 on the surface of these cells by FACS analysis using specific antibodies (Fig. 3A). Siglec-5 is widely expressed in monocyte and in neutrophils, with some variability in these latter cells. Siglec-14 is expressed in neutrophils while Siglec-9 was not detected in either cell type. Intriguingly, NadA could not be detected in any tested condition and only a very faint shift was revealed in monocytes from donor 1 (Fig. 3B). Although we do not explain these data, it will be argued in the discussion.

### NadA binding to Siglec-5 and Siglec-14 on eukaryotic membrane requires V-set domain

**Fig 4:**
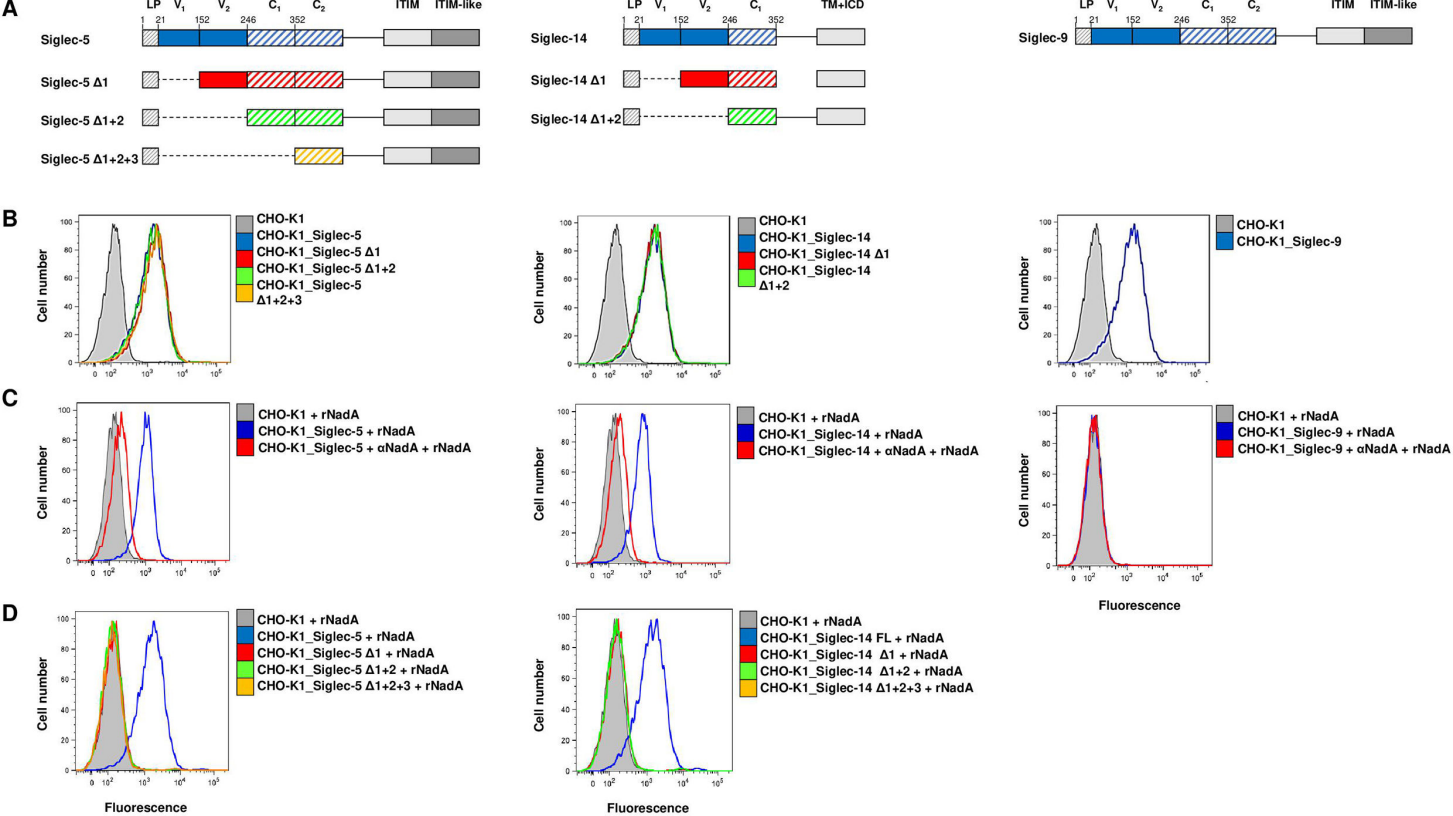
Binding of recombinant soluble NadA to Siglec-5 and Siglec-14 expressed in CHO-K1 cell membrane. (**A**) Schematic representation of Siglec full-length and truncated constructs used to transfect eukaryotic cells. Full-length Siglec-5, Siglec-14, and Siglec-9 are colored in blue. The Siglec-5 and Siglec-14 lacking the V_1_ set domain (Δ1) (Siglec-5_Δ20–151_ and Siglec-14_Δ20–151_) are represented in red, Siglec-5 and Siglec-14 lacking the V_1_ and V_2_ set domains (Δ1 + 2) (Siglec-5_Δ20–245_ and Siglec-14_Δ20–245_) are illustrated in green, and Siglec-5 lacking the V_1_ and V_2_ set domains and the C_1_ set domain (Δ1 + 2 + 3) (Siglec-5_Δ20–351_) is schematized in orange. LP = leader peptide; V_1_ and V_2_ = V-set Ig domains; C_1_ and C_2_ = C-set Ig domains; TM + ICD = transmembrane and intracellular domains. (**B**) Siglecs' surface exposure of all the constructs on CHO-K1 transfected cells was assessed via FACS. Colored curves represent the surface exposure of different Siglec constructs, while gray profiles represent untransfected CHO-K1 cells. (**C**) rNadA binding to CHO-K1 cells expressing Siglec-5, Siglec-14, and Siglec-9 was shown by FACS (blue profiles). Tinted gray curves represent the negative control (non-transfected CHO-K1 cells incubated with rNadA). Red curves illustrate the inhibition of the binding on transfected CHO-K1 cells preincubated with polyclonal anti-receptor antibodies and extensive washed prior to rNadA incubation. (**D**) rNadA binding on transfected (colored profiles) and non-transfected (tinted gray curves) CHO-K1 cells was assess by FACS using mouse polyclonal anti-NadA antibodies. Plots are representative of three independent experiments performed in triplicate.

In the absence of a consistent NadA binding to monocytes and neutrophils, we sought to have evidence of the Siglec recognition on the membrane of eukaryotic cells by overexpressing the sialic acid receptors. Thus, we transiently transfected CHO-K1 cells individually with Siglec-5, Siglec-14, and the control Siglec-9, to be able to verify the binding of the adhesin and its inhibition by anti-Siglec antibodies. In parallel, we sought to identify the NadA binding domain on the two Siglecs by expressing deletion mutants of Siglec-5 and Siglec-14. The deletion mutants were obtained by trimming one by one the extracellular domains of the Siglec-5 and Siglec-14 resulting in three and two truncated constructs for each Siglec, respectively (Fig. 4A). Firstly, we confirmed the expression of Siglecs on the surface of CHO-K1 cells by FACS analysis using rabbit anti-Siglec antibodies (Fig. 4B). Then, FACS analysis performed incubating CHO-K1 expressing full-length Siglecs with rNadA and using a polyclonal anti-NadA serum evidenced that rNadA exclusively bound eukaryotic cells that exposed either Siglec-5 or Siglec-14 on their surface and not to untransfected cells or to cells expressing Siglec-9 (Fig. 4C, blue curves). Furthermore, preincubation of transfected cells with polyclonal anti-receptor antibodies prior to rNadA incubation inhibited the binding to Siglec-5 and Siglec-14 (Fig. 4C, red curves). Finally, to identify the specific domain responsible for interaction of Siglecs with NadA, we incubated CHO-K1 cells expressing Siglec-5 and Siglec-14 deleted proteins with rNadA and the binding revealed by FACS analysis. Deletion of the first N-terminal domain both in cases of Sigle-5 and Siglec-14 completely abrogated NadA binding (Fig. 4D). Taken together, these data assessed that the intact Siglec V-set domain, which is the recognition domain of sialic acids (25), is also responsible for the interaction with rNadA.

### Siglec-5 and Siglec-14, but also Siglec-9, bind NadA on a bacterial membrane context

**Fig 5:**
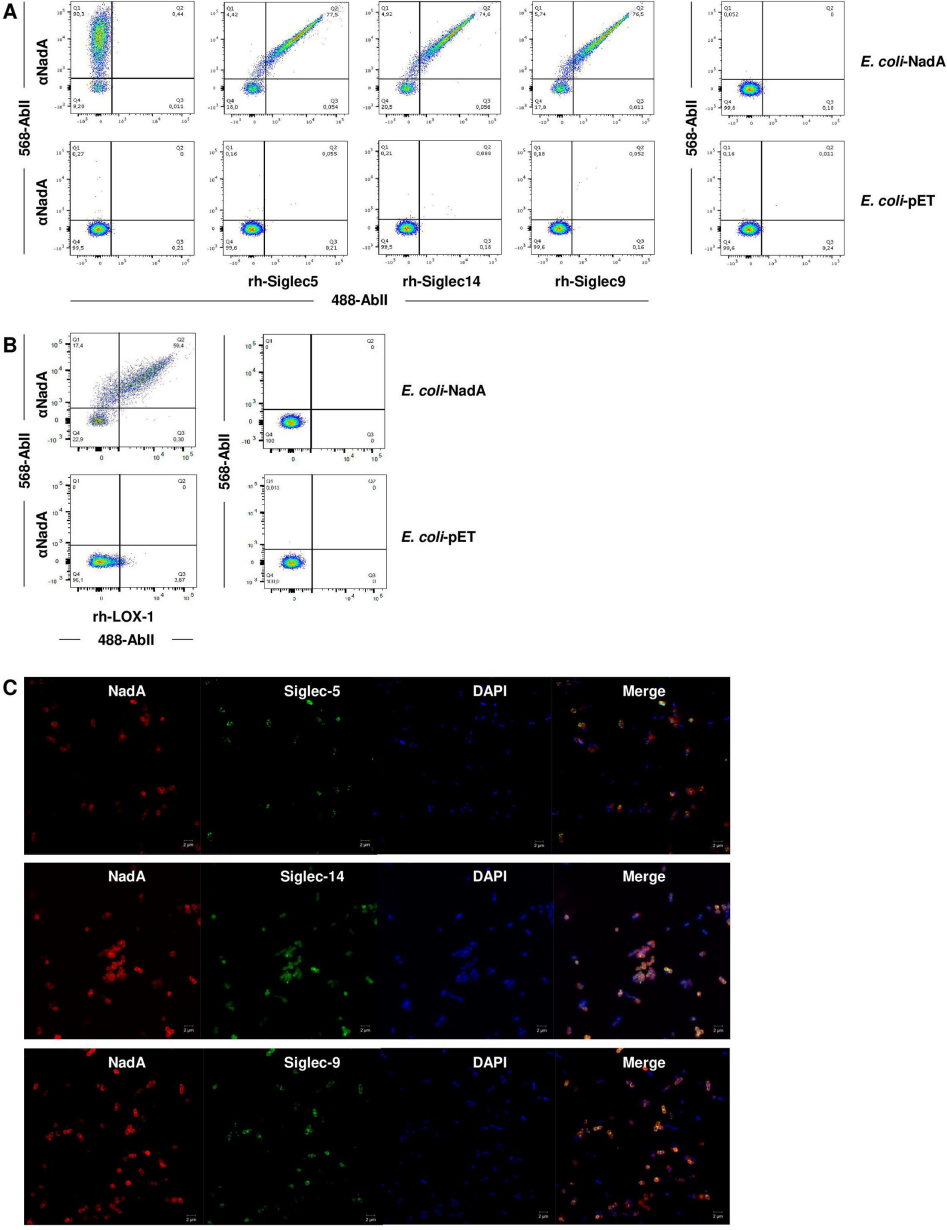
Binding of soluble Siglec-5 and Siglec-14 to *E. coli*-NadA. Binding of rh-Siglec-5, rh-Siglec-14, rh-Siglec-9, and rh-LOX-1 to *E. coli-*NadA was revealed by flow cytometry (**A and B**) and confocal microscopy (**C**). *E. coli-*NadA (A and B, top panels) and *E. coli-*pET (A and B, bottom panels) bacteria were incubated with 1µg/mL of Siglec-5, Siglec-14, Siglec-9, and rh-LOX-1 prior to the incubation with polyclonal anti-NadA sera. Siglec-NadA complexes were revealed by 488-anti-human and 568-anti-mouse secondary antibodies, while Siglec-LOX interaction was revealed with 488-anti-rabbit and 568-anti-mouse secondary antibodies. Bacteria incubated with secondary antibodies alone were used as negative controls, while bacteria stained exclusively with anti-NadA sera were used to verify the expression of NadA. (**C**) For confocal microscopy *E. coli*-NadA cells were stained as for FACS analysis and then immobilized on polylysine-coated glass slide. DAPI was used to stain bacterial chromosome (blue). Scale bars represent 2 µm.

Once assessed that rNadA was able to recognize Siglec-5 and Siglec-14 expressed on non-permissive eukaryotic cells, we sought to investigate whether recombinant Siglec-5 and Siglec-14 could bind directly to the surface of a *E. coli* BL21(DE3)T1 strain expressing full-length NadA (*E. coli*-NadA). Indeed, it has been previously shown that NadA expressed in *E. coli* is exposed on the membrane mimicking native meningococcal NadA (14, 18). Binding of the three rh-Siglecs to *E. coli*-NadA was investigated both by FACS and confocal performing co-staining analyses (Fig. 5). *E. coli* cells were simultaneously stained with differently labeled secondary fluorescent anti-NadA and anti-rh-Siglec antibodies to evaluate the colocalization of the interactors. NadA surface expression in *E. coli*-NadA cells was not homogenous (roughly 20% of cells seem to be NadA negative; Fig. 5A and B, Q4) possibly due to the overloaded status of the folding machinery/trafficking systems ascribable to the high NadA expression (42, 43). However, Siglec-5 and Siglec-14, along with the positive control rh-LOX-1, specifically bound *E. coli* cells in a NadA-dependent manner (Fig. 5A and B). Indeed, control bacteria represented by *E. coli*-pET cells transformed with a vector void of any insert (Fig. 5A and B, bottom right panels) were negative. Unexpectedly, also, Siglec-9 was found associated to NadA expressing bacteria (Fig. 5A and C). These results were not coherent with our analysis performed with the recombinant protein, suggesting that NadA expressed in a membrane context may recruit additional Siglecs with different mechanisms.

### *Neisseria meningitidis* serogroup B binds Siglec-5, Siglec-14, and Siglec-9 through NadA

**Fig 6:**
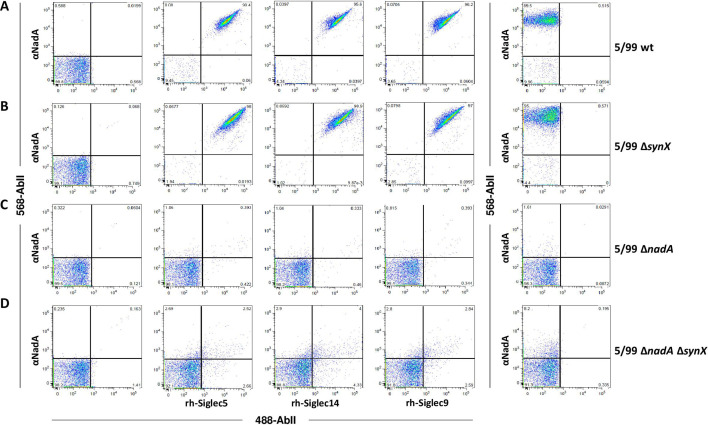
Binding of rh-Siglec-5, rh-Siglec-14, and rh-Siglec-9 to *N. meningitidis* 5/99 wt and acapsulated strains. Co-staining FACS analyses of *N. meningitidis* 5/99 wt (A), 5/99 *ΔsynX* (B), 5/99 Δ*nadA* (C), and 5/99 Δ*synx* Δ*nadA* (D) strains incubated with rh-Siglecs (10µg/mL). After incubation, bacteria were stained with polyclonal anti-NadA antibodies. The interaction between Siglec-NadA was revealed by 488-anti-human and 568-anti-mouse secondary antibodies. Bacteria incubated with secondary antibodies alone were used as negative controls, while bacteria stained exclusively with anti-NadA sera were used to verify the expression of NadA.

To further confirm the binding of Siglecs to surface exposed NadA, we checked this interaction directly on live *Neisseria meningitidis* serogroup B strains by FACS surface co-staining experiments. The 5/99 meningococcal strain was selected for the analysis since it naturally expresses higher levels of NadA with respect to other isolates (7). Moreover, considering that binding of Siglec-5 to sialylated LPS has been reported on acapsulated MC58 strain (44), we generated also a capsule deficient strain by knocking out the *synX* gene in the 5/99 strain, with the aim to verify if a NadA-independent binding of Siglecs occurred in the isolate under investigation. Specific *nadA* deletion mutants were then generated in both genetic backgrounds as control. FACS analysis performed on the four strains (Fig. 6) showed that Siglec-5, Siglec-14, and Siglec-9 recognized the surface of 5/99 wt and 5/99 Δ*synX* strains expressing NadA, whereas they did not bind the strains deprived of NadA (5/99 Δ*nadA* and 5/99 Δ*synX* Δ*nadA*). Interestingly, 5/99 wt and 5/99 Δ*synX* surface co-staining experiments revealed superimposable results suggesting that sialic acids of the 5/99 capsule did not bind Siglecs. Therefore, the results confirmed no difference in the NadA-dependent binding of all three Siglecs on a bacterial membrane context.

All together, these experiments confirmed NadA-dependent binding of Siglec-5 and Siglec-14, but they also revealed that soluble Siglec-9 ectodomain did bind to NadA exposed on meningococci.

### Adhesion/invasion of *Neisseria meningitidis* serogroup B to CHO-K1 cells expressing Siglec-5 and Siglec-14 is NadA dependent

**Fig 7:**
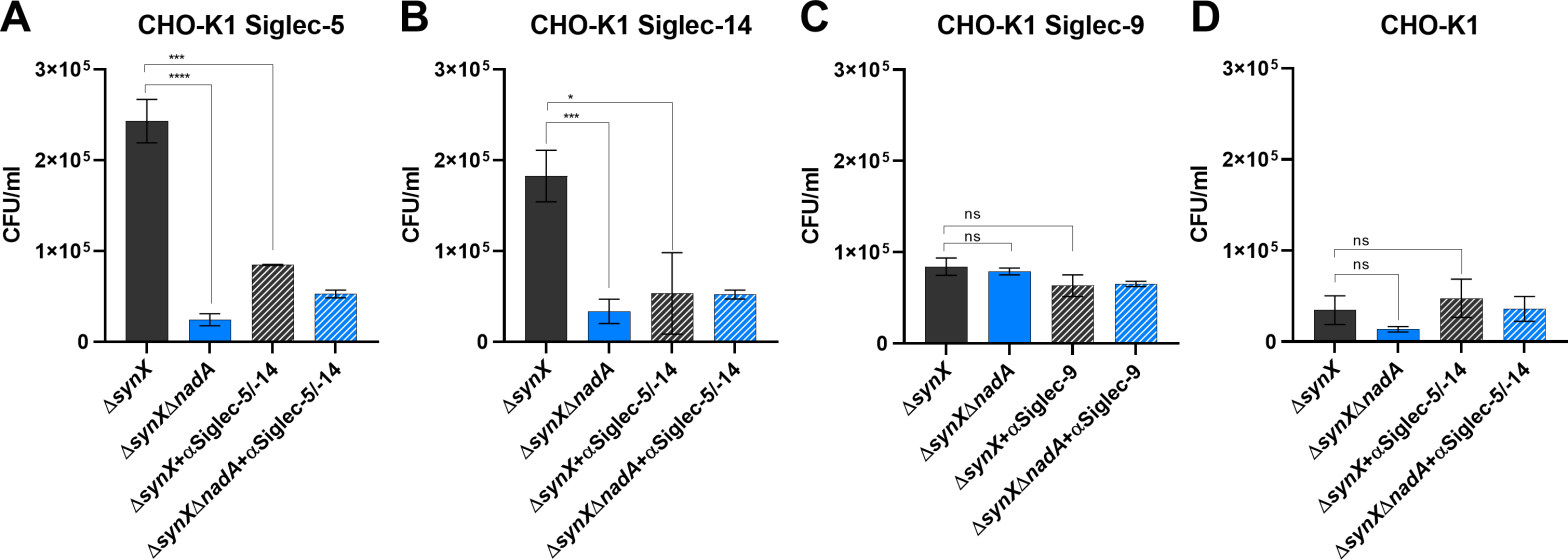
Adhesion/invasion assay of *N. meningitidis* to CHO-K1 cells expressing Siglecs. Monolayers of CHO-K1 cells transfected to express Siglec-5 (**A**), Siglec-14 (**B**), Siglec-9 (**C**), and empty vectors (**D**) were infected with 5/99 Δ*synX* (dark gray) or 5/99 Δ*synX* Δ*nadA* (blue) strains. The number of adhered/invaded bacteria was determined by CFU counting assay. Inhibition of bacterial adhesion was performed by preincubating cells with 10µg/mL of antibodies against Siglecs (stripped bars). Results are reported as CFU/mL. Error bars represents mean and±SEM of four experiments. Statistical analysis was performed using one-way ANOVA with Tukey’s post test (*P < 0.05, ***P < 0.001, ****P < 0.0001, and ns, *P* > 0.05)..

Since the binding of recombinant Siglec-9 to bacterial membrane exposed NadA (*E. coli* and MenB) was very puzzling, we investigated whether Siglec-9 versus Siglec-5 and Siglec-14 binding had a different biological outcome. We used transiently transfected CHO-K1 cells individually expressing Siglec-5, Siglec-14, and Siglec-9 to verify NadA-driven adhesion/invasion on 5/99 Δ*synX* and 5/99 Δ*synX* Δ*nadA*. We used the acapsulated (Δ*synX*) bacteria to eliminate the anti-adherent properties of the capsule that masks adhesins/invasins (45, 46). Cells transfected with empty vector were used as control. After 3 h of infection at 37°C, cells were lysed and CFU were counted to assess the ability of the bacteria to adhere/invade CHO-K1 cells expressing Siglecs. In parallel, cells were also pretreated with anti-Siglec-5, Siglec-14, or Siglec-9 antibodies to assess the specificity of the NadA binding to the putative receptors. As shown in Fig. 7, a significant higher number of NadA expressing bacteria (5/99 Δ*synX*) were recovered into CHO-K1 cells expressing both Siglec-5 and Siglec-14 (Fig. 7A and B, respectively), whereas cells transfected with Siglec-9 and empty vector (Fig. 7C and D, respectively) retained lower number of CFU like bacterial strains lacking NadA (5/99 Δ*synX* Δ*nadA*). Indeed, we did not find any statistical difference in recovered CFU from CHO-K1 cells transfected with empty vector or Siglec-9 and infected with 5/99 Δ*synX* and 5/99 Δ*synX* Δ*nadA*. These results suggested that Siglec-5 and Siglec-14 behaved as NadA putative receptors and mediated a biological effect, whereas Siglec-9 did not. This conclusion was also corroborated by the strong reduction of recovered bacteria obtained following pretreatment of cells with Siglec-5 and Siglec-14 antibodies, which did not occur when cells were incubated with anti-Siglec-9 antibodies (Fig. 7). This experiment is consistent with a NadA-mediated binding to the membrane receptors Siglec-5 and Siglec-14 and suggested that *N. meningitidis* may exploit these receptors to adhere to and invade host cells. In contrast, Siglec-9 did not appear to have any receptor-like activity on bacteria carrying NadA at least in mediating adhesion/invasion processes.

## DISCUSSION

Herein, we have performed an unbiased protein microarray screening approach described previously by Scietti et al. (20), with an expanded library of human secretome proteins (37). Recombinant human Siglec-5 and Siglec-14 turned out to have a high score of NadA recognition then confirmed by the high affinity *K*_*D*_ of 4.05 and 5.40 nM determined for Siglec-14 and Siglec-5, respectively. The precise binding site has here been resolved by HDX-MS, which is a powerful tool to accurately locate protein regions involved in the association between interactors (47). Indeed, the HDX rate of protein backbone amides depends on the stability of their hydrogen bonding network with the solvent (48–50), which can be altered, usually reduced, upon ligand binding. By HDX-MS experiments, we have determined the conformational impact of Siglec-5 interaction on NadA in the coiled-coil head domain of NadA and its connecting segment to the wings (A24–F51 and V76–K82). This finding agrees with the evidence that NadA_140–342_, which lacks the head domain, was not able to associate to Siglec-5 and Siglec-14 in SPR. Since the wings do not display significant conformational effects, we hypothesized that the elongated structure of the lectin is able to insert in the highly solvent and ligand accessible pocket between the head and the wings of NadA, potentially altering the wings orientation.

Our findings confirmed and refined the results from previous works, based on the usage of deletion mutants and inhibition of antibody binding, which indicated the head as responsible for the binding to host receptors and adhesion/entry (14, 51). Indeed, it appears that the peculiar structure of the N-terminus, which does not show structural homology to any other known adhesin (10, 11), mediates recognition to several candidate receptors. Liguori et al. defined critical residues for Lox-1 binding in an intermonomer crevice and that the NadA-Lox-1 complex hence formed competed with two potent neutralizing antibodies targeting the same region of the head (10). To our knowledge, among proteins so far identified to bind Siglecs in a sialic acid-independent manner, NadA is the only one whose binding site has been defined at such a structural level.

Siglecs are best known for their crucial role in fine tuning the immune and inflammatory responses by recognizing precise patterns of cell surface carbohydrate moieties (23, 52). More recent studies have highlighted engagement of these molecules in infection diseases (31). Pathogens have developed the ability to interfere with this defense mechanism usually wearing mimetic glycans but also expressing protein that can directly bind to Siglecs, such as the β-protein of group B *Streptococcus* (53) and Porin B from *Neisseria gonorrhoeae* (54). All these interactions lead to consequences to the diseases caused by these pathogens. Most of the Siglecs transmit an inhibitory signal following engagement with substrates. Appearance of paired receptors has been inferred to be due to the necessity of balancing this inhibitory signal exploited by pathogens (30). Thus, engagement of Siglec-5 unleashes an inhibitory signal through recruitment of the cellular phosphatases SHIP-1 and SHIP-2 (Src-homology SH2 domain containing phosphatase) recruited on its intracellular ITIM (23). Vice versa, the intramembrane region of Siglec-14 interacts with the adaptor proteins DAP10/DAP12 upon activation allowing association on the ITAM motif of the spleen tyrosine kinase (syk) that triggers activation of the cells (29). Intriguingly, previous works have shown that the binding of NadA or *E. coli*-NadA with primary monocytes, derived macrophages, and dendritic cells induces Th1 differentiation and proinflammatory signaling (15–17), suggesting that NadA targets Siglec-14. In our hands, the binding of recombinant NadA to isolated human monocytes or neutrophil cells was poorly or not detectable by cytofluorimetric analysis despite binding was reported by other authors (16). However, it is to note an important technical difference between our and their experimental setting. Mazzon et al. used a fluorescent labeled NadA that was directly detectable by FACS while we used unlabeled NadA, mouse anti-NadA serum, and anti-mouse fluorescent label secondary antibody. This procedure needed a preliminary Fcγ receptor blocking procedure achieved by incubating cells with human serum. It is possible that, during this step, myeloid cells capture factors that then hinder recognition of Siglecs by NadA. It is also to notice that monocytes used in the abovementioned report (16) were isolated from donors' buffy coats, thus a representative population of healthy people. It has been proved that, in a significant percentage of the human population, the gene coding for Siglec-14 is deleted leading to difference in immune response to pathogens (55, 56). This polymorphism-dependent response has been studied for the β-protein of group B *Streptococcus* (GBS) that binds to Siglec-5 and Siglec-14 in a sialic acid independent manner. In the heterozygote, a stimulatory response following Siglec-14 engagement activates the p38 MAP kinase and AKT axis facilitating bacterial clearance and inducing proinflammatory response. Indeed, isolated monocytes and neutrophils from *Siglec14*-null donors show an impaired response to GBS and a dominance of the inhibitory pathways of SHP-1 and SHP-2 recruitment triggered by the bacterial β-protein binding to Siglec-5 (56). Indeed, it would be very interesting to verify if the NadA induced response is dependent on genotype or even on the pattern of expressed Siglecs or cell surface glycans, in turn, variable following genetic and environmental factors (57). These topics deserves further studies and working is ongoing to elucidate these features. However, when expressed in a heterologous system, both Siglec-5 and Siglec-14, but not Siglec-9, bind NadA at the same extent and anti-receptor antibodies antagonize this interaction.

To our knowledge, this is the first report that identified two candidates showing functional features of NadA receptors linking its biochemical features to biological activity. A previous report identified β1-integrin as epithelial cell receptors (19). However, in their study, the authors did not find evidence of NadA-driven β1-dependent binding or entry of the acapsulated *N. meningitidis* serogroup B MC58 strain and that of the NadA-deprived isogenic mutant. Their conclusion was that NadA can use other still unidentified receptors (19). Integrins are heterodimeric proteins; thus, its eventually binding to NadA could not had been revealed with our approach since the array included only monomeric species.

Ultimately, although the most extensive studies on the adhesion and invasion features of NadA have been conducted on Chang cells, derived from the epithelial of human conjunctiva (ATCC CCL-20.2) (8, 13, 14, 18, 19), NadA remains still orphan of a robust epithelial cellular receptor candidate nor our screening was able to identify any species. The high affinity binding between NadA and Lox-1 (20) candidates this protein as receptor on endothelial cells. Nevertheless, to date, no functional evidence of such activity or NadA-driven biological outcome on this cell type have been reported. However, the identification of several NadA interactors expressed on different cell types suggests that the adhesin may have still unrecognized functions in *N. meningitidis* pathogenesis. Although binding to Siglec-5 and Siglec-14 on myeloid cells indicates a role for NadA in modulating immune response, other functions can be hypothesized. Following engagement, Siglecs are internalized and this feature could be exploited as a route of entry to create an intracellular niche to survive *in vivo*. Although *N. meningitidis* is not an intracellular bacterium, a recent report on *Streptococcus pneumoniae*, another pathogen not known to have an intracellular survival, pointed out that specialized cells may represent a reservoir for bacteria spreading into the host (58). Specifically, after both the mouse and pig challenges, streptococci are quickly cleared from all tissues except for the spleen where they colonize marginal zone metallophilic macrophages and replicate intracellularly. Spreading to neighborhood cells starts 8 h after inoculation, ending with dissemination in blood and septicemia. Macrophage invasion was driven by recognition of the host sialic acids, but invasion was mediated by Siglec-1 (sialoadhesin, CD169) and mouse treatment with anti-Siglec-1 antibody prevented cellular invasion leading to clearance of streptococcus (58). Such a peculiar way of invasion is challenging to be revealed especially for pathogens with no animal model of infection such as *Neisseria meningitidis*. However, sialylated strains of *Neisseria meningitidis* have been found to bind Siglec-1 and Siglec-5 promoting internalization (44). Siglecs exhibit affinity constants for sialic acids in the millimolar range (25), 3–4 order of magnitude lower than NadA. We speculate that the sialic acids incorporated into the meningococcus capsule or LOS are involved in an initial tethering step by low affinity binding to Siglecs then replaced by a tighter NadA-Siglec-5 or Siglec-14 binding that could lead to invasion.

The most intriguing result from our work has been the binding of Siglec-9 to NadA expressed on the membrane of homologous and heterologous bacteria and not to the recombinant adhesin. Although we do not have a satisfying explanation for this finding, our recent data could provide an explanation (59). A comparative HDX analysis performed on rNadA and on outer membrane vesicles carrying full length NadA had revealed that the adhesin exists in two different conformations, a “closed” trimer and an “open” trimer, likely dynamically exchanging. The full length NadA anchored to the membrane shows higher abundance of the open trimer conformation compared to rNadA, which is conversely mostly present in the closed conformation. Thus, we hypothesize that these conformational changes, membrane anchor dependent, may allow NadA to recruit additional factors. Also, it could also be that the β-barrel anchor of the full length adhesin may be involved in this recruitment providing that the predicted external loops include hydrophilic residues and should be oriented on the external surface. In any case, the NadA interaction to Siglec-5 and Siglec-14 on one side, and Siglec-9 on the other, does not have the same biological outcome: the former promoted adhesion/invasion while the latter did not. Differential binding specificity of a sialic acid independent binding toward Siglecs has also been found for the *Neisseria gonorrhea* Porin B (54), In the context of the whole bacteria, this porin was found to bind to chimpanzees Siglec-5 and Siglec-14 and in one strain to Siglec-9 despite the recombinant proteins did not or very poorly (54). Although we were not able to fully shed light on the interaction of membrane NadA with Siglec-9, the implication of NadA binding to receptors of the immune responses, like Siglec-14 and Siglec-5, leads us to hypothesize that the role of NadA goes further than the exclusive epithelial tissue invasion.

## MATERIALS AND METHODS

### Protein microarray construction, processing, and data analysis

Protein microarrays were generated by spotting approximately 2,846 humans and 297 mice, His-tag or His and Fc-tag recombinant soluble proteins (from a 0.5 mg/mL solution in a final glycerol concentration of 40%) produced at the Genomic Institute of the Novartis Research Foundation (GNF) as described in reference 37. Most of the proteins belonged to separate preparations and were spotted in two or more copies. Protein spotting, printing controls, and data analysis have been performed as described in reference 20; rNadA_24–350_ was diluted in Nap-PBS at final concentration of 1 µM and overlaid on the arrays for 1 h. After washing, arrays were incubated with rabbit polyclonal serum raised against NadA (diluted 1:2,000) at RT for 1 h and then revealed with an AlexaFluor 647-conjugated anti-rabbit IgG secondary antibody (Jackson Immunoresearch). For each protein, the mean fluorescence intensity (MFI) of replicated spots was determined, after subtraction of the background value surrounding each spot. As control, a microarray was run in parallel and it was probed with the Alexa Fluor 647-conjugated anti-rabbit IgG alone to determine any non-specific binding to the printed proteins. Proteins with a high coefficient of variation between the two replicates were discarded. All obtained MFI scores were classified in four categories: (i) high reactivity: MFI ≥ 30,000; (ii) medium reactivity: 15,000 ≤ MFI < 30,000; (iii) low reactivity: 5,000 ≤ MFI < 15,000; and (iv) no reactivity MFI < 5,000.

### Recombinant proteins

Fc tagged recombinant human Siglec-9, Siglec-14, and Siglec-5 were purchased at R&D Systems (1139-SL, 4905-SL, and 1072-SL, respectively). Herein we will refer to them as rh-Siglec-5, rh-Siglec-14, and rh-Siglec-9.

NadA V3 represents the full-length molecule (crystal structure of NadA_24-170_ -PDB: 6EUN). Three *nadA* gene fragments corresponding to the full length ectodomain lacking the leader peptide and the anchor domain (rNadA or NadA_24–350_), the trimeric “head domain” containing also part of the stalk (NadA_24–170_), and the “stalk domain” lacking the amino-terminal region (NadA_140–342_) were cloned by PCR from *N. meningitidis* strain 2996, using the polymerase incomplete primer extension (PIPE) method (10, 60). The amplified fragments were cloned into a pET-21 vector (Novagen), enabling cytoplasmic expression of the NadA construct with a C-terminal 6×His tag. Recombinant proteins were expressed and purified as previously reported (10).

### Surface plasmon resonance

#### Kinetics analysis

Experiment was performed on a Biacore T200 instrument (GE Healthcare). The recombinant NadA ectodomain was immobilized on a CM5 Series two sensor chip (GE Healthcare) using the standard amine coupling procedure applying the protein to the activated sensor surface at 100 µg/mL and in 10 mM sodium acetate, pH 4.5. At the end, about 3,000 RU was captured. Single cycle kinetics were performed using HSB-EP+ (10 mM HEPES, pH 7.4, 0.15 M NaCl, 3 mM EDTA, 0.005% [vol/vol] Surfactant P20) as running buffer, Fc-tagged recombinant human Siglec proteins were injected at 25°C using 60 s intervals at 6.25, 12.5, 25, 50, and 100 nM at 40 µL/min and for 60 s each. Dissociation was followed for 1,200 s. Using the Biacore Evaluation software version 3.0, curves were fitted using the two-state binding model of interaction and kinetic constants were determined.

#### Binding analysis

Experiment was performed on a Biacore 8K instrument (GE Healthcare). The recombinant NadA ectodomain, head domain, and stalk domain were immobilized on a CM5 Series S two sensor chip (GE Healthcare) using the standard amine coupling procedure. In the case of thermally stressed NadA, the protein was heated at 80°C for 2 h prior to the immobilization onto the chip. The recombinant NadA was applied to the activated sensor surface at 100 µg/mL and in 10 mM sodium acetate, pH 4.5, while the head and stalk domains were used at the concentration of 50 µg/mL in 10 mM sodium acetate, pH 5.0 and pH 4.5, respectively. About 3,000, 2,600, and 930 RU were captured for the rNadA protein, head domain, and stalk domain, respectively. The binding analysis was performed using HSB-EP+ as running buffer. Fc-tagged recombinant Siglec proteins were injected at 25°C for 180 s, at concentration of 100 nM at 30 µL/min. Dissociation was followed for 300 s. Biacore Insight Evaluation software was used to determine the binding responses. Graphs in Fig. 1 represent sensorgrams (red) and fitted curves (black) after injection of Siglecs at increasing concentrations. Curve fitting in both cases was performed using the two-state binding model. For each sensorgram, blank subtraction was performed by subtracting the signal obtained with injection of buffer alone instead of Siglec proteins. Plot abscissae represent time in seconds, while ordinates represent the binding response measured in resonance units (RU).

#### Hydrogen and deuterium exchange mass spectrometry (HDX-MS) analysis

##### NadA peptide identification

Peptide identification was performed by LC-MS^E^ analysis of peptides generated upon on-line pepsin digestion of non-deuterated rNadA. Samples were subjected to LC separation identical to that performed for labelled samples. The acquired MS/MS data were processed with ProteinLynx Global Server (PLGS) version 3.0 (Waters). DynamX version 3.0 (Waters) was used to filter peptides by selecting those fragmented at least two times, presenting 0.2 fragments per amino acid, and found in at least four out of five acquired LC-MS runs. Additionally, the MS trace of the identified peptides was visually inspected and peptides with insufficient signal-to-noise intensity were discarded.

##### Deuterium labeling at room temperature and on ice

rNadA was incubated alone or in the presence of Siglec-5 (NadA:Siglec-5 ratio is 1:3.8) for 30 min at room temperature, in order to allow the complex to form prior labeling. HDX was then initiated by 4.17-fold dilution with deuterated PBS buffer (at 95% D_2_O), yielding to 72.2% of final deuterium content in the reaction mixture. At various time intervals (15 s, 1 min, 10 min, 30 min, 100 min, and 1,000 min), an aliquot, containing 12.8 pmol of NadA, was withdrawn and quenched 1:1 with ice-cold 300 mM phosphate buffer at 4 M urea (final pH/D 2.5). After quenching, samples were immediately frozen in liquid nitrogen and stored at −80°C until LC-MS analysis. The labeling for 30 min was performed two additional times for the state “NadA alone,” producing triplicates for that time point. An analogous experimental procedure was carried out for deuterium labelling on ice. After the complex equilibrated on ice, HDX was initiated by dilution into ice-cold deuterated buffer and the reaction quenched after 4 s, 15 s, 30 s, and 1 min. The labeling for 1 min was performed two additional times for both states; thus, triplicate samples were also performed.

##### Maximally labeled control

rNadA was diluted in Solvent A (0.23% formic acid), injected onto the LC system, and digested on-line. Peptides eluting from the pepsin column were collected and the solvent evaporated to dryness. Peptides were resuspended in deuterated PBS buffer at 72.2% of deuterium fraction, as in the protein labelling mixture, and exchanged was allowed for 6 h at 30°C. The reaction was quenched 1:1 with ice-cold 300 mM phosphate buffer at 4 M urea (final pH/D 2.5). The sample was immediately frozen in liquid nitrogen and stored at −80°C until LC-MS analysis.

##### Liquid chromatography-mass spectrometry method

Frozen quenched samples were rapidly thawed with a table-top centrifuge and injected onto a refrigerated UPLC system (NanoAcquity, Waters) with all chromatographic elements held at 0°C. The samples passed through an in-house packed column containing pepsin immobilized on agarose resin beads at 20°C, except for the maximally labelled sample. The generated peptides were trapped on a Vanguard column (BEH C18, 130  Å, 1.7  µm, 2.1 mm × 5 mm; Waters) and desalted for 2.5  min in solvent A (0.23% formic acid in MilliQ water, pH 2.5) at a flow rate of 200  µL/min. Subsequently, the peptides were separated over an Acquity UPLC column (C18, 130  Å, 1.7  µm, 1.0 mm × 100  mm; Waters), with a 9-min linear gradient rising from 8% to 40% of solvent B (0.23% formic acid in acetonitrile, pH 2.5) at 40  µL/min. Following the chromatographic separation, the peptides were analyzed using a hybrid ESI-Q-TOF mass spectrometer (Synapt G2-Si, Waters). The MS was set in positive ionization mode with spray voltage of 3 kV and the ions were further separated by ion mobility for enhancing peak capacity. The MS spectra were acquired in range from 50 to 2,000 *m*/*z*, performing a scan every 0.5 s. Human Glu-fibrinopeptide (Sigma-Aldrich) served as an internal standard and the peptide signal was recorded throughout all the analysis.

##### Data analysis and statistical approach

DynamX 3.0 was used to calculate deuterium incorporation at peptide level. The differential deuterium content of overlapping peptides was used to localize the protein regions impacted by the ligand binding. The statistical significance of a difference in HDX between two states was determined by calculating a confidence interval (CI) based on the pooled standard deviation of the peptide deuterium uptake (SD) in time points performed in triplicates following Weis' recommendations (61, 62). For each state, the SDs were pooled using the root mean square, according to equation 1:


(1) $${SD}_{state}=\sqrt{\frac{\sum {{SD}_{i}}^{2}}{N}}$$

where *N* is the number of peptides considered. A pooled SD for the states performed in triplicates was subsequently calculated using equation 2 or 3 for the experiment on ice and at room temperature, respectively:


(2) $${SD}_{pool}=\sqrt{{{SD}_{NadA\;alone}}^{2}+{{SD}_{NadA/Siglec-5}}^{2}}$$

(3) $${SD}_{pool}=\sqrt{{{SD}_{NadA\;alone}}^{2}\times 2}$$

The pooled SD was used to calculate the CI at significance level of 98% with a zero-centered average difference in deuterium uptake, considering a two-tailed distribution with two degrees of freedom, by using equation 4


(4) $$CI=\mp 6.965\times \frac{{SD}_{pool}}{\sqrt{n}}$$

where *n* is the number of replicates performed, equal to 3.

### Isolation of human monocytes and neutrophils

Monocytes were enriched by negative selection using Isolation Kit from StemCell Technologies (Vancouver, BC, Canada) according to the manufacturer's instructions. Briefly, antibody-coated microbeads, targeting cell surface antigens on human blood cells (CD2, CD3, CD19, CD20, CD56, CD66b, CD123, and glycophorin A) and dextran, were used for magnetic separation of unwanted population of PBMC. Unbound cells (monocytes) were collected in PBS buffer containing 2% FBS (HS; Sigma-Aldrich Inc., USA) and 1 mM EDTA.

Neutrophiles were isolated from healty donors' blood following Surewaard et al. procedure (63). Briefly, blood diluted 1:1 in PBS was loaded on a dual layer ficoll gradient (1.119 g/mL and 1.077 g/mL) and centrifuged at 20 min at 396 × g in a swinging bucket rotor, 22°C without braking. The top layer of ficoll (yellow) containing plasma and PBMC, and the second layer (white) were aspired. The mixture of PMN and erythrocytes was washed with 50 mL of RPMI-HSA and centrifuged for 10 min at 249 × g at 4°C. Erythrocytes were lysed by hypotonic shock by resuspending the pellet in 9 mL of cold sterile deionized H_2_O for exactly 30 s before adding 1 mL of 10 × PBS to restore osmolarity. Cells were washed again with RPMI-HSA at 4°C for 10 min at 249 × g and finally resuspended in RPMI-HSA.

### Siglec expression and NadA binding on human monocytes and neutrophiles

Siglec expression was assessed by FACS using anti-Siglec-5 polyclonal antibody produced in goat (PA5-47058), anti-Siglec-14 polyclonal produced in rabbit (SAB 1303894), and anti-Siglec-9 polyclonal antibody produced in rabbit (PA5-11683). Secondary antibodies used were Alexa-488 chicken anti-goat IgG (H+L) cross-adsorbed secondary antibody (No. A-21467) and goat anti-rabbit IgG (H+L) cross-adsorbed secondary antibody (A-11008). To avoid nonspecific binding from Fc receptors, cells were incubated on ice for 20 min in a solution of human serum diluted 1:50 in sterile PBS. Approximately 5 × 10^5^ monocytes and 1 × 10^6^ neutrophiles per well were seeded in a 96-well plate round bottom and incubated 30 min at 37°C in PBS + 1% BSA with 10 µM of cytochalasin D, to avoid internalization of Siglec receptor.

To detect NadA binding, we mixed cells with 200 µg/mL rNadA or blocking solution (PBS + 1% BSA) alone for 30 min at 37°C in 5% CO_2_. Cells were then washed and incubated with mouse 9F11 monoclonal anti-NadA (diluted 1:1,000) for 30 min at 4°C, and with allophycocyanin (APC)-conjugated goat F(ab)_2_ antibody to mouse Ig (diluted 1:100; Thermo Scientific) for 30 min at 4°C. Cells were then analyzed with a FACS-Scan flow cytometer (Beckton-Dickinson). The mean fluorescence intensity (MFI) for each population was calculated using FlowJo software (version 9.8; Tree Star, Inc, Ashland, OR). Control curves (gray in Fig. 3) represent the secondary antibodies alone (αGoat AbII, αRabbit AbII, and αNadA AbII).

### Cloning and expression of Siglecs in CHO-K1 cells

Chinese hamster ovary CHO-K1 cells (ATCC CCL-61) were grown in F-12K medium supplemented with 100 U/mL penicillin, 100 mg/mL streptomycin, and 10% FBS and maintained at 37°C in a controlled humidified atmosphere containing 5% CO_2_. Siglec expressions were achieved by transfecting the cells using Lipofectamine LTX Reagent with PLUS Reagent (Life Technologies) according to the manufacturer's instructions. The codon optimized full-length coding regions of Siglec-5 and Siglec-14 (GenBank accession no. NP_003821.1 and NP_001092082.1, respectively) were obtained from GeneCopoeia as pEZ-M14 FLAG-tag vectors. The codon optimized full-length coding sequence of Siglec-9 (GenBank accession no. NP_055256.1) was obtained by GeneArt as pcDNA3.1(+) His-tag vector. PIPE method was used to generate sequential sub-domain deletions (V-set and/or C2-set domains) and to obtain the following truncated receptors: Siglec-5 Δ1 (Siglec-5_Δ20–151_), Siglec-5 Δ1 + 2 (Siglec-5_Δ20–245_), Siglec-5 Δ1 + 2 + 3 (Siglec-5_Δ20–351_), Siglec-14 Δ1 (Siglec-14_Δ20–151_), and Siglec-14 Δ1 + 2 (Siglec-14_Δ20–245_).

Primers used to generate these constructs are listed in Table 2.

**TABLE 2:** Primed used to generate Siglec deletion mutants

Primer	Sequence	Specification
Rev 5&14	TGGCTTCTCCTGCAG	Siglec-5 and -14 common reverse primer
Fw 5&14 Δ1	CTGCAGGAGAAGCCAGCCCTGATAGAGAAACCC	Deletion domain 1 on Siglec-5 and -14
Fw 5 Δ1+2	CTGCAGGAGAAGCCAGTCTCCTATGCACCACAG	Deletion domains 1 and 2 on Siglec-5
Fw 14 Δ1+2	CTGCAGGAGAAGCCAGTCTCCTATGCTCCACAG	Deletion domains 1 and 2 on Siglec-14
Fw 5 Δ1+2+3	CTGCAGGAGAAGCCACTCTCAGTTTACTCCCTC	Deletion domains 1, 2, and 3 on Siglec-5

### Flow cytometry of rNadA binding to transfected CHO-K1 cells

Recombinant NadA binding assay was performed as previously described (8). Briefly, untreated or transfected cells were non-enzymatically detached using cell dissociation solution (CDS; Sigma), harvested, and suspended in RPMI-1640 (Gibco) medium supplemented with 1% FBS. Siglec membrane expression was assessed by FACS using anti-Siglec-5 polyclonal antibody produced in rabbit (SAB1303597; Sigma), anti-Siglec-14 polyclonal antibody produced in rabbit (SAB1303894; Sigma), and anti-Siglec-9 polyclonal antibody produced in rabbit (PA5-11683; Invitrogen). The secondary antibody used was APC-conjugated chicken F(ab)_2_ antibody to rabbit Ig.

To detect rNadA binding, we mixed approximately 3 × 10^5^ cells with 200 µg/mL rNadA or blocking solution (PBS + 1% FBS) alone for 30 min at 37°C in 5% CO_2_. Cells were then washed and incubated with NadA mouse polyclonal antiserum (diluted 1:1,000) for 1 h at 4°C, and with allophycocyanin (APC)-conjugated goat F(ab)_2_ antibody to mouse Ig (diluted 1:100; Thermo Scientific) for 30 min at 4°C. The inhibition of NadA binding was performed by preincubating cells with 50 µg/mL of each specific anti-Siglec polyclonal antibodies in FBS-free medium for 30 min at 37°C in 5% CO_2_ prior to rNadA addition. Cells were then analyzed with a FACS-Scan flow cytometer (Beckton-Dickinson). The mean fluorescence intensity (MFI) for each population was calculated using FlowJo software (version 9.8; Tree Star, Inc, Ashland, OR). In Fig. 4, tinted grey curves represent the negative control. Plots are representative of three independent experiments performed in triplicate.

### Animal immunization

Anti-NadA mouse sera were obtained immunizing sixteen 8-week-old CD1 female mice (Charles River Laboratories International, Inc, Wilmington, MA, USA) with 20 µg of NadA. The antigen was administered intraperitoneally on days 1, 21, and 35, together with aluminum hydroxide (3 mg/mL) as adjuvant. Sera were collected 2 weeks after the third dose and pooled. Protocol was approved by the Italian Ministry of Health (Approval Number AWB 201704). All mice were housed under specific pathogen-free conditions at the GSK Vaccines Animal Resource Center in compliance with the relevant guidelines (Italian Legislative Decree No. 26/2014). To prepare rabbit antisera, we used 50 µg of NadA to immunize six 9-week-old New Zealand female rabbits (Charles River Laboratories International, Inc, Wilmington, MA, USA). The antigen was administered subcutaneously on days 1, 21, and 35, together with aluminum hydroxide (3 mg/mL) as adjuvant. Sera were collected 2 weeks after the third dose and pooled.

### Expression of NadA in *E. coli* cells, FACS, and confocal surface staining

*Escherichia coli* BL21(DE3) T1 (New England BioLabs) cells were used to express the full‐length NadA protein or void vector as previously described (14). Surface protein expression of NadA full-length was achieved without addition of IPTG and exploiting the leakage of the promoter.

For FACS surface staining, bacteria were resuspended in PBS + 1% BSA and incubated with human rh-Siglecs or rh-LOX-1 (1798LX-050; R&D Systems) to a final concentration of 1 µg/mL. After 30 min of incubation with gentle mixing at 37°C, bacteria were washed extensively with PBS + 1% BSA and then incubated with mouse NadA antisera (1:400). The Siglec-NadA complex was then revealed with Alexa Fluor 488 goat anti-human IgG (H+L) (Invitrogen, A11013) and Alexa Fluor 568 goat anti-mouse IgG (H+L) (Invitrogen, A11004) diluted 1:400, while LOX-1/Siglec interaction was revealed with rabbit LOX-1 polyclonal antibody (Thermo Fisher; PA5-109203, diluted 1:100) followed with Alexa Fluor 488 goat anti-rabbit IgG (H+L) (Invitrogen, A11013) plus Alexa Fluor 568 goat anti-mouse IgG (H+L) (Invitrogen, A11004) both diluted 1:400. Bacteria were fixed in 0.5% formaldehyde and fluorescence was recorded with SOS II (BD Bioscience) acquiring 10,000 events. Data were analyzed using Flow-Jo v.3 (FlowJo, LLC). Bacteria incubated with secondary antibodies alone were used as negative controls.

For confocal microscopy, cells were treated as described above. Bacteria were spread on polylysine-coated slides (Thermo Scientific). Labelled preparations were mounted with ProLong Gold antifade reagent with DAPI (Invitrogen), analysed with a Zeiss LSM-710 confocal microscope, and images were captured using ZEN software (Carl Zeiss).

### *Neisseria meningitidis* 5/99 strain mutants

*Neisseria meningitidis* strain 5/99 was routinely grown on GC agar (Difco) or Mueller Hinton (MH) agar (Difco) at 37°C plus 5% CO_2_. For liquid cultures, overnight growth was used to inoculate GC or MH broth. When required, erythromycin and chloramphenicol were added to achieve final concentrations of 5 µg/mL. The *nadA* knock out 5/99 strain is described in reference 64.

To generate acapsulated *N. meningitidis* 5/99 strain, the *synX* flanking regions were amplified by PCR from the chromosomal DNA of strain NZ98/254 using the primer pairs SynX_upF XmaI/SynX_upR XbaI to amplify the 3′ flanking region and SynX_doF NsiI/SynX_doR SpeI to amplify the 5′ flanking region, respectively. Obtained fragments were then ligated with the chloramphenicol cassette, amplified using the primer pair ClmR_xba_fw/ClmR_Nsi_rev from pSLComClmR (65), and cloned between XmaI and SpeI restriction sites of pSL1190 vector. Prior to transformation into *N. meningitidis* 5/99 or 5/99 Δ*nadA* strains, plasmids were linearized by restriction digestion using SpeI (New England Biolabs) following the manufacturer’s instructions. For transformation of naturally competent *N. meningitidis*, five to ten freshly colonies grown overnight were re-suspended in 30 µL of PBS and spotted onto GC agar plates. A total of 1–10 μg of linearized plasmid DNA was added, allowed to dry and incubated for 5–6 h at 37°C. Transformants were then selected on GC agar plates containing erythromycin or chloramphenicol after overnight incubation at 37°C. SynX deletion was verified by PCR analysis using primers Synx_ext_F and Synx_ext_R to check the correct insertion of the resistance marker by a double homologous recombination. NadA deletion was verified by Western blot.

Primers used to generate these mutants are listed in the Table 3.

**TABLE 3:** Primers used to generate *Neisseria meningitidis* mutants

Primer	Sequence
Synx_upF XmaI	GCGTCCCGGGTCAGCGGCATACGCACAC
Synx_upR XbaI	GTCCGTCTAGAGAAGTCGGCTCTGGTACC
Synx_doF NsiI	GTCGATGCATCGCACCAGCACAGAAAGA
Synx_doR_2 SpeI	GTCGACTAGTCATCAAATAGAGAAAAAGCTTCACG
ClmR_xba_fw	ATTCGTGTACAAGTCAACCGTGATATAGATTAAAAGTGG
ClmR_Nsi_rev	ATTCGGCATCGGATCCACGCGTCTTAAGGCG
Synx_ext_F	CGTACTACATTGCCACGTGTC
Synx_ext_R	GCCTGAGGTAATTGTTGGCG

### Meningococcal FACS surface staining

*N. meningitidis* 5/99 wt, 5/99 Δ*nadA,* 5/99 Δ*synX*, and 5/99 Δ*nadA* Δ*synX* strains were cultured overnight on GC agar medium with Kellogg’s supplement I (66) at 37°C in an atmosphere of 5% CO_2_. The following day, bacteria were grown under the same conditions in MH broth plus 0.25% glucose until early exponential phase.

For FACS surface staining, bacteria were resuspended in PBS + 1% BSA and incubated with 10 µg/mL of rh-Siglecs for 30min at 37°C. Bacteria were washed extensively with PBS + 1% BSA and then incubated with mouse NadA antisera (1:400). Alexa Fluor 488 goat anti-human IgG (H+L) (Invitrogen, A11013) and Alexa Fluor 568 goat anti-mouse IgG (H+L) (Invitrogen, A11004) diluted 1:400 were used to detect Siglec-NadA complex. Bacteria were fixed in 0.5% formaldehyde and fluorescence was recorded with SOS II (BD Bioscience). Data were analysed using Flow-Jo v.3 (FlowJo, LLC). Bacteria incubated with secondary antibodies alone were used as negative controls.

### Adhesion and inhibition assay

A total of 1.5 × 10^5^ CHO-K1 cells were seeded onto 24-well tissue culture plates and transfected as previously described. At least 2 h before infection protocol, medium was changed with fresh infection medium (IM; F-12K medium with 1% FBS, antibiotic free). For inhibition assay, antibodies anti-Siglec-5/14 (R&D Systems) and anti-Siglec-9 (Sigma) were added 20 min before challenge/incubation with bacteria. *N. meningitidis* strains were grown in GC medium until optical density = 0.5 (about 2.5 h), pelleted and resuspended in infection medium (MOI = 200:1). Bacterial suspension was added to the apical surface of the cultures for 3 h at 37°C and 5% CO_2._ After removal of non-adherent bacteria by extensive washings, cells were lysed with 1% saponin and serial dilutions of the suspension were plated onto GC agar to count the CFU. Data in Fig. 7 were graphed using GraphPad Prism 6.04 software (GraphPad Software) and show mean and standard deviation. Statistical analysis was performed using one-way ANOVA with Tukey’s post test (* *P* < 0.05, ***P < 0.001, ****P < 0.0001, and ns, *P* > 0.05).

## SUPPLEMENTAL MATERIAL

Fig. S1 mbio.01107-24-s0001.pdf Deuterium uptake plots of of peptides.

Fig. S2 mbio.01107-24-s0002.pdf Deuterium uptake plots of peptides.

Fig S3 mbio.01107-24-s0003.pdf Summary of HDX effects in NadA head domain.

Legends mbio.01107-24-s0004.docx Legends to supplemental material.

Table S1 mbio.01107-24-s0005.xlsx Complete list of proteins used for the array.

Table S2 mbio.01107-24-s0006.xlsx HDX-MS analysis on rNadA.

Table S3 mbio.01107-24-s0007.xlsx HDX-MS analysis on rNadA in complex with Siglec-5.

ASM does not own the copyrights to Supplemental Material that may be linked to, or accessed through, an article. The authors have granted ASM a non-exclusive, world-wide license to publish the Supplemental Material files. Please contact the corresponding author directly for reuse.
