# Supplementary material for: Neisserial adhesin A (NadA) binds human Siglec-5 and Siglec-14 with high affinity and promotes bacterial adhesion/invasion
Source: mBio. 2024 Jul 23;15(8):e01107-24. doi: 10.1128/mbio.01107-24 (PMC11323535; doi:10.1128/mbio.01107-24)
Supplement: Fig. S1 — Deuterium uptake plots of of peptides. [file mbio.01107-24-s0001.pdf]

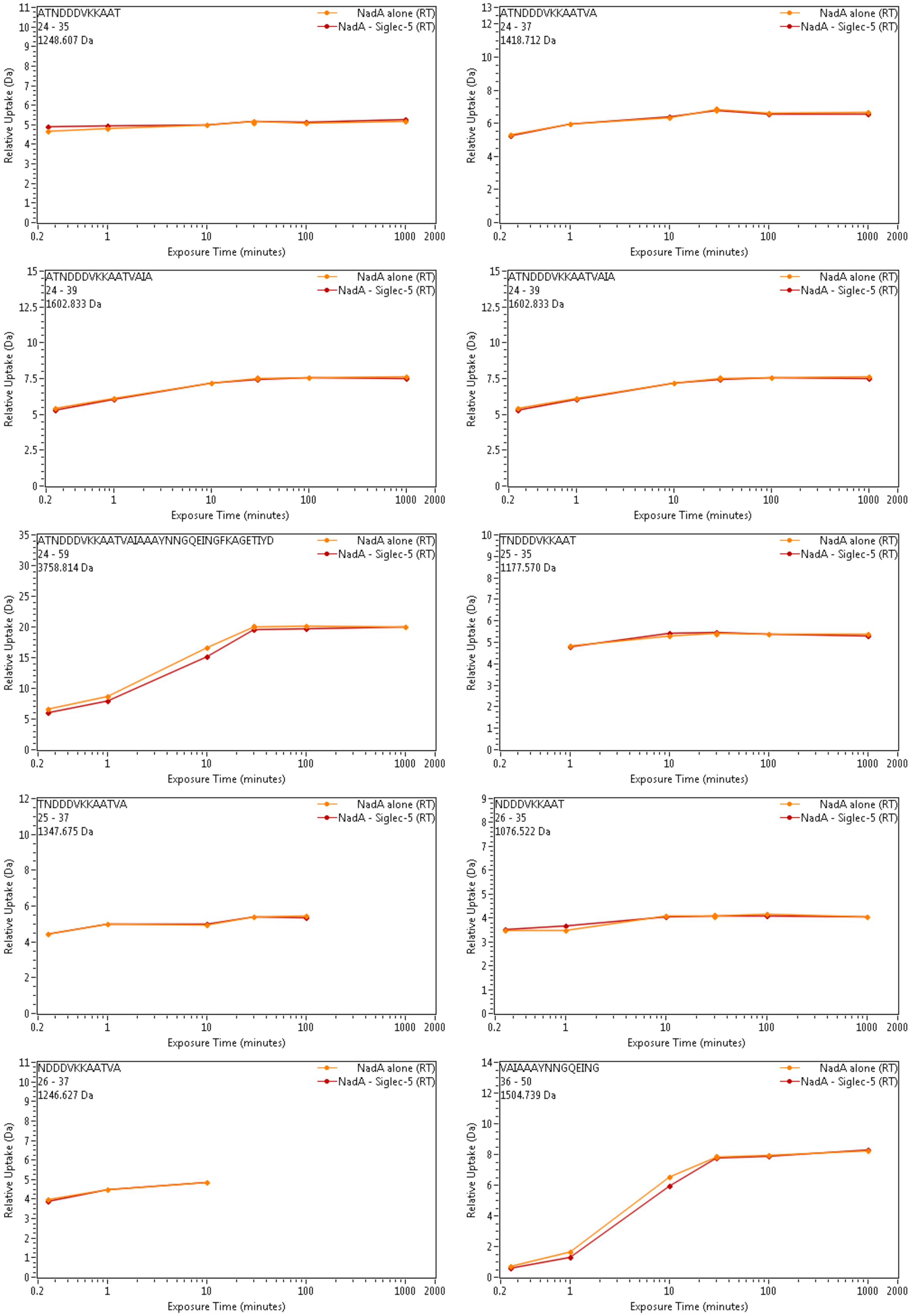
ATNDDDVKKAAT
24 - 35
1248.607 Da
ATNDDDVKKAATVA
24 - 37
1418.712 Da
ATNDDDVKKAATVAIA
24 - 39
1602.833 Da
ATNDDDVKKAATVAIA
24 - 39
1602.833 Da
ATNDDDVKKAATVAIAAAYNNGQEINGFKAGETIYD
24 - 59
3758.814 Da
TNDDDVKKAAT
25 - 35
1177.570 Da
TNDDDVKKAATVA
25 - 37
1347.675 Da
NDDDVKKAAT
26 - 35
1076.522 Da
NDDDVKKAATVA
26 - 37
1246.627 Da
VAIAAAYNNGQEING
36 - 50
1504.739 Da
NadA alone (RT)
NadA - Siglec-5 (RT)
Relative Uptake (Da)
Exposure Time (minutes)

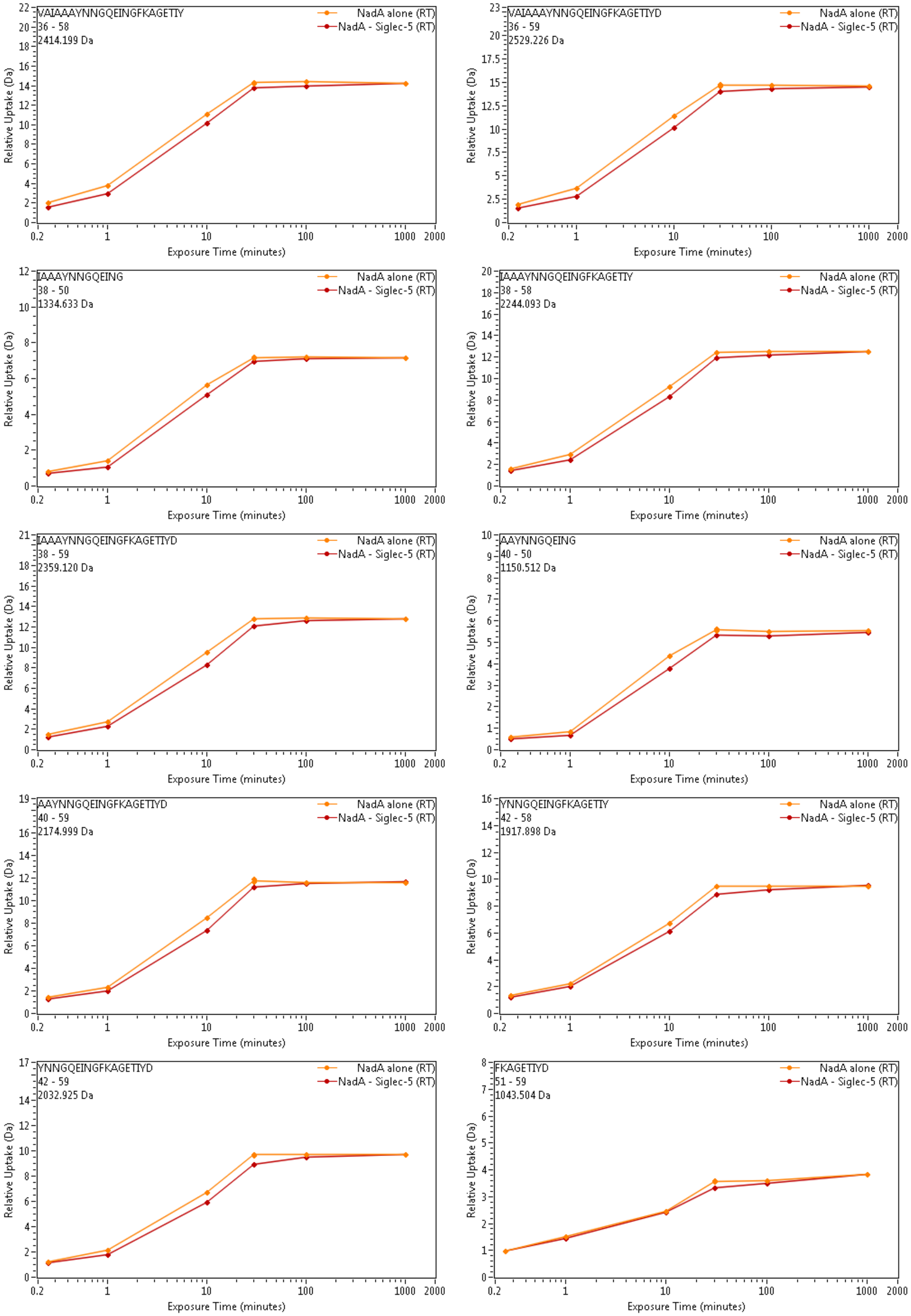
VAIAAAYNNGQEINGFKAGETIY
36 - 58
2414.199 Da
VAIAAAYNNGQEINGFKAGETIYD
36 - 59
2529.226 Da
IAAAYNNGQEING
38 - 50
1334.633 Da
IAAAYNNGQEINGFKAGETIY
38 - 58
2244.093 Da
IAAAYNNGQEINGFKAGETIYD
38 - 59
2359.120 Da
AAYNNGQEING
40 - 50
1150.512 Da
AAYNNGQEINGFKAGETIYD
40 - 59
2174.999 Da
YNNGQEINGFKAGETIY
42 - 58
1917.898 Da
YNNGQEINGFKAGETIYD
42 - 59
2032.925 Da
FKAGETIYD
51 - 59
1043.504 Da
NadA alone (RT)
NadA - Siglec-5 (RT)
Relative Uptake (Da)
Exposure Time (minutes)

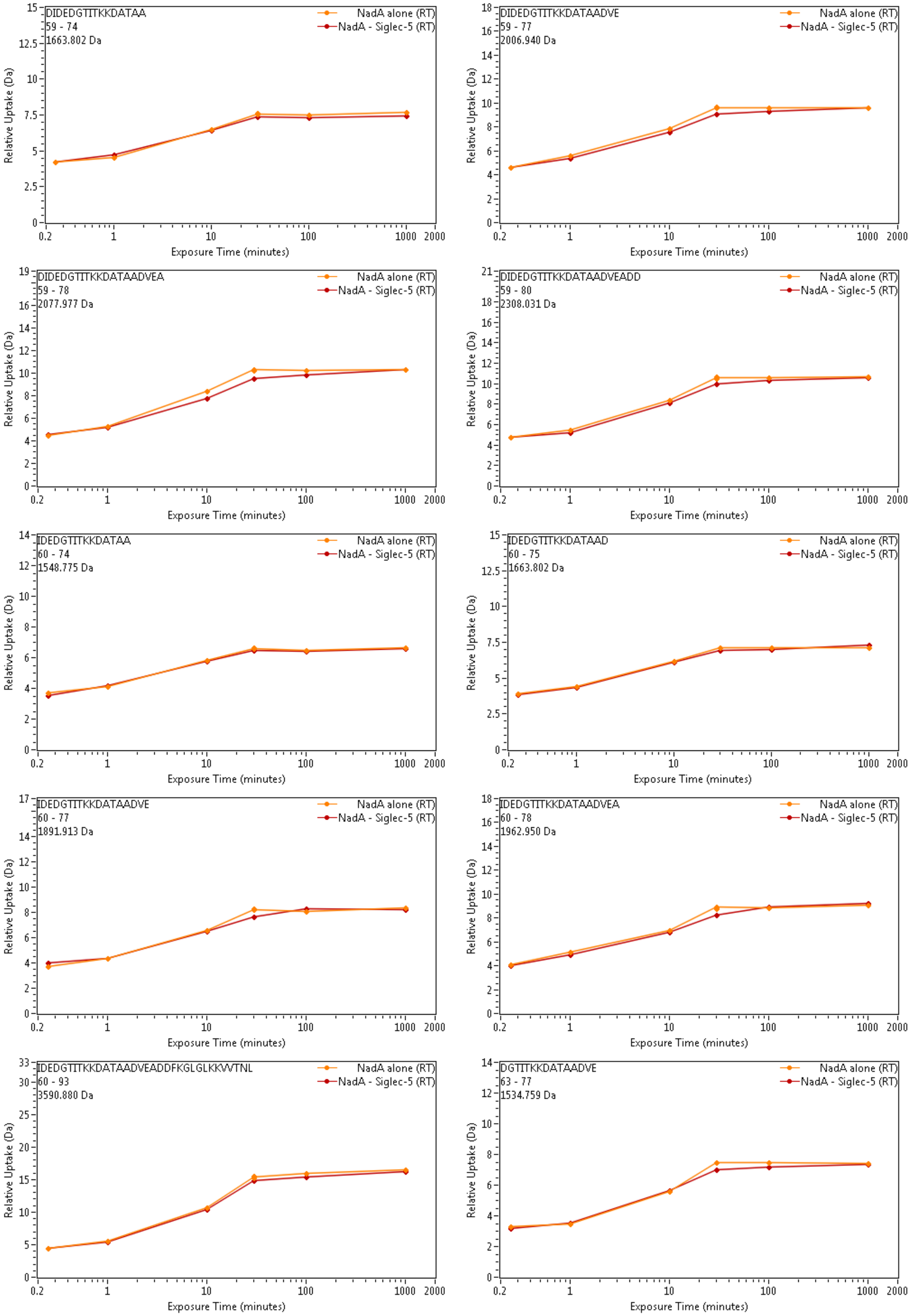
DIDEDGTITKKDATAA
59 - 74
1663.802 Da
DIDEDGTITKKDATAADVE
59 - 77
2006.940 Da
DIDEDGTITKKDATAADVEA
59 - 78
2077.977 Da
DIDEDGTITKKDATAADVEADD
59 - 80
2308.031 Da
IDEDGTITKKDATAA
60 - 74
1548.775 Da
IDEDGTITKKDATAAD
60 - 75
1663.802 Da
IDEDGTITKKDATAADVE
60 - 77
1891.913 Da
IDEDGTITKKDATAADVEA
60 - 78
1962.950 Da
IDEDGTITKKDATAADVEADDFKGLGLKKVVTNL
60 - 93
3590.880 Da
DGTITKKDATAADVE
63 - 77
1534.759 Da
NadA alone (RT)
NadA - Siglec-5 (RT)
Relative Uptake (Da)
Exposure Time (minutes)
0.2
1
10
100
1000
2000

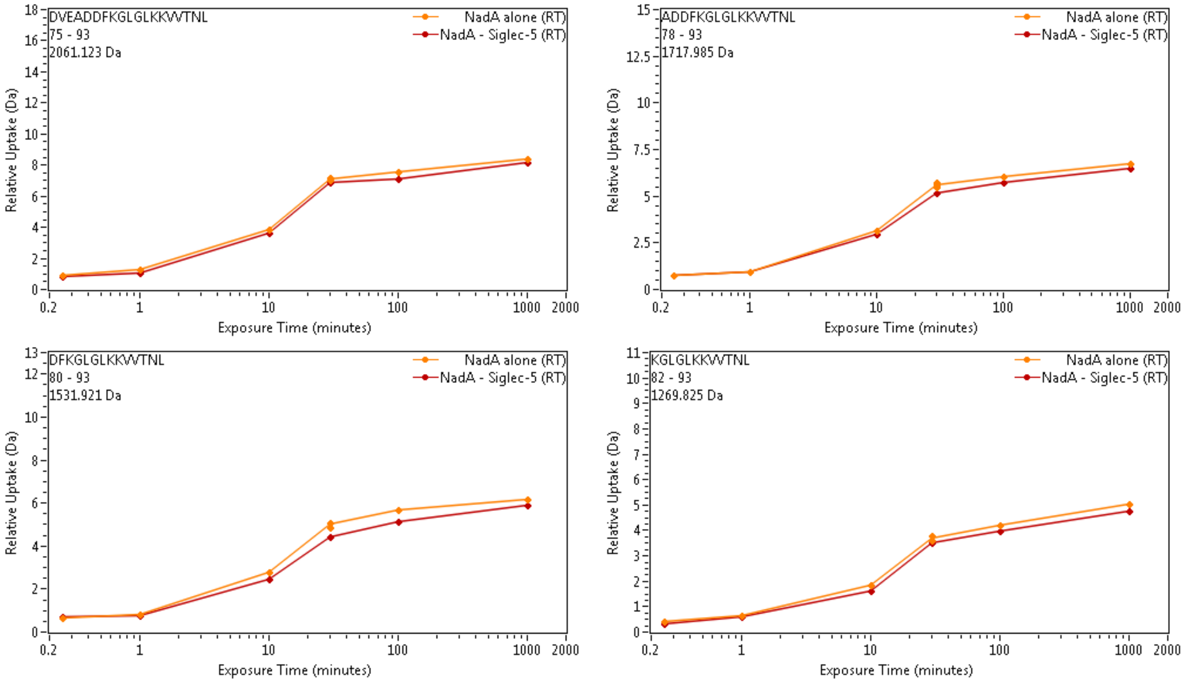

Fig. S1. Deuterium uptake plots of peptides spanning residues 24-93 as exported from DynamX 3.0, showing the relative deuterium content upon labelling at RT. On the x-axis, the exposure time to the deuterated buffer (minutes); on the y-axis, the deuterium uptake Da).
