# Supplementary figures and images for: Neisserial adhesin A (NadA) binds human Siglec-5 and Siglec-14 with high affinity and promotes bacterial adhesion/invasion
Source: mBio. 2024 Jul 23;15(8):e01107-24. doi: 10.1128/mbio.01107-24 (PMC11323535; doi:10.1128/mbio.01107-24)

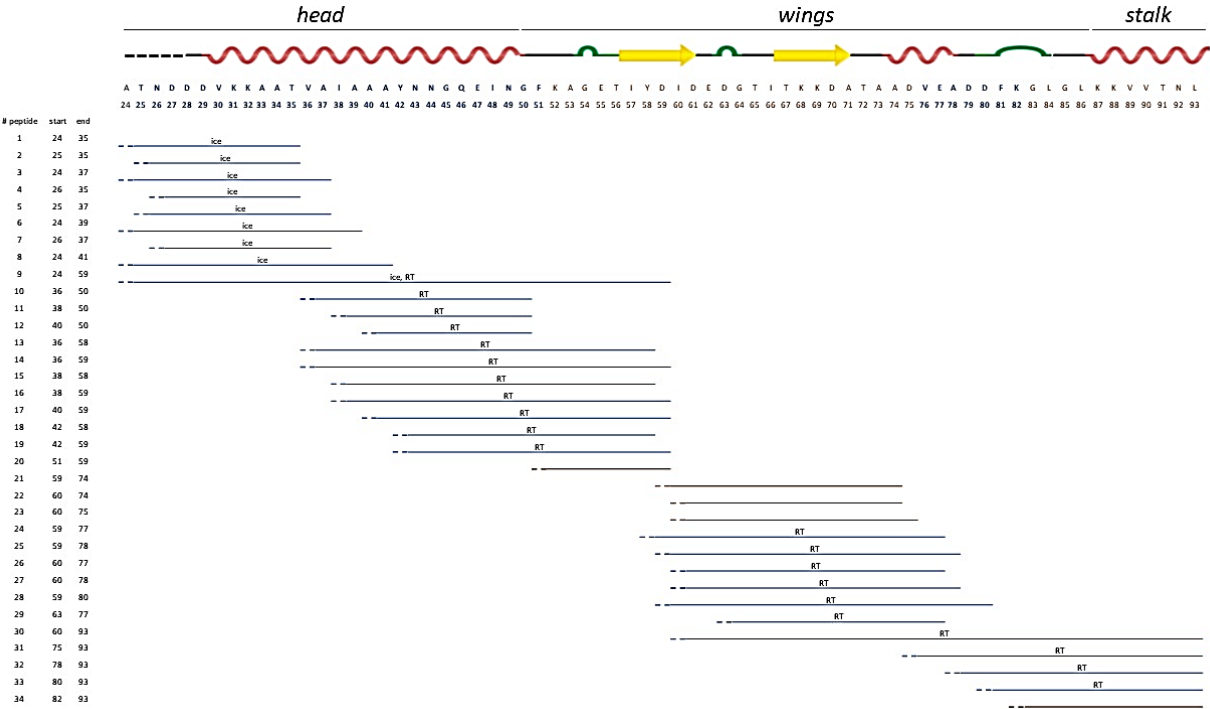

Supplement: Fig. S2 — Deuterium uptake plots of peptides. [file mbio.01107-24-s0002.pdf]

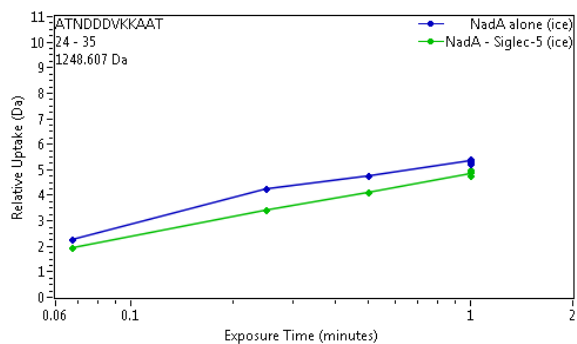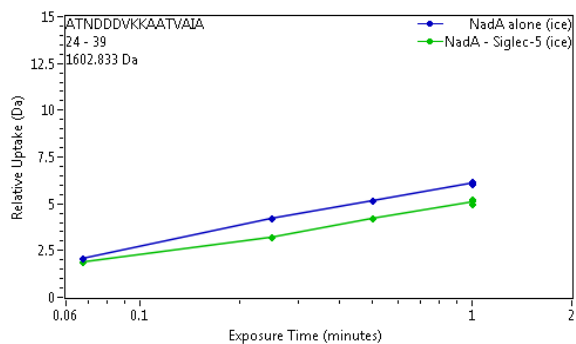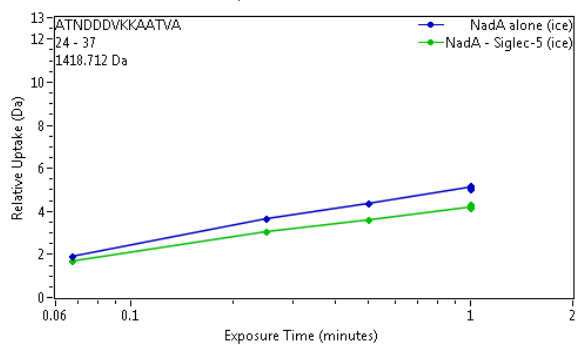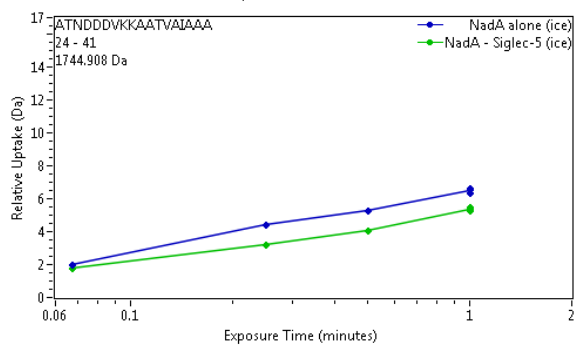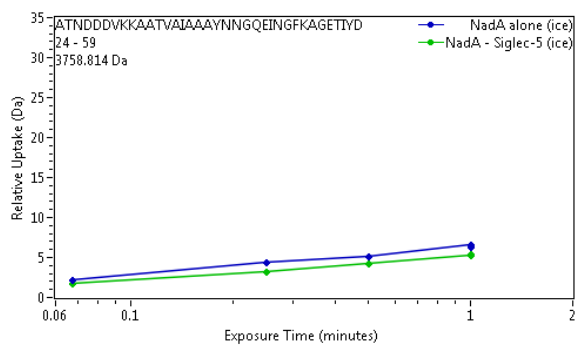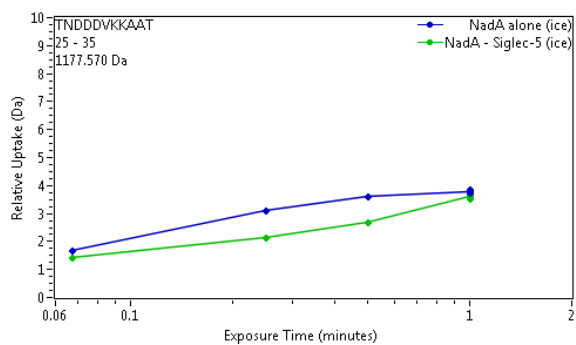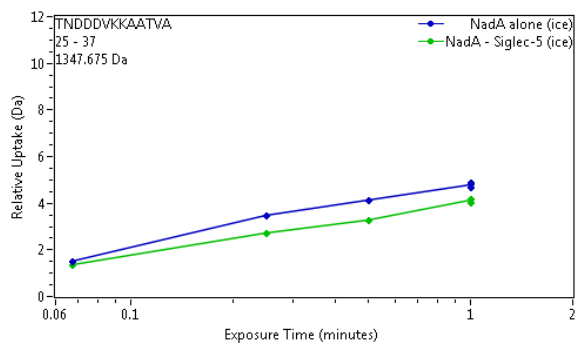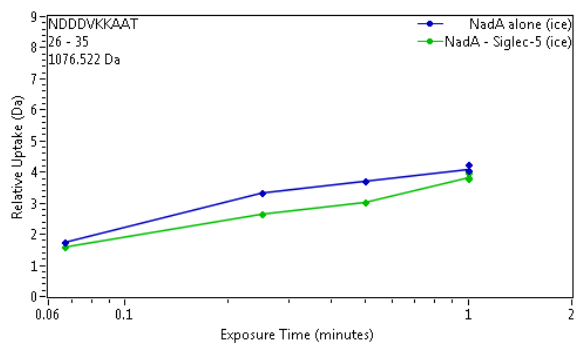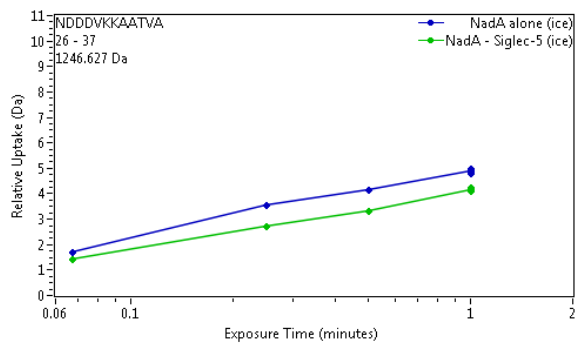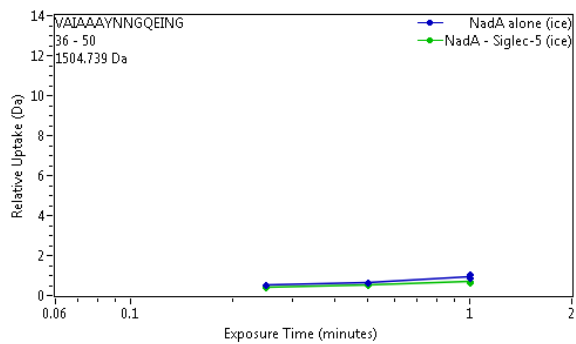

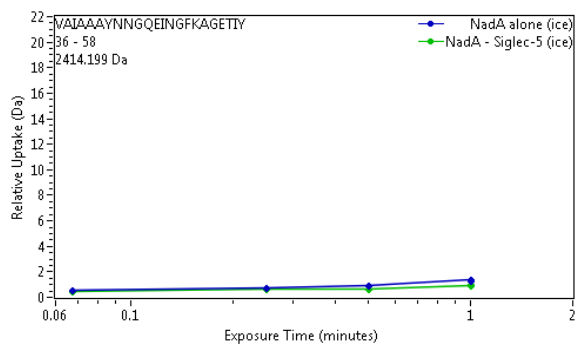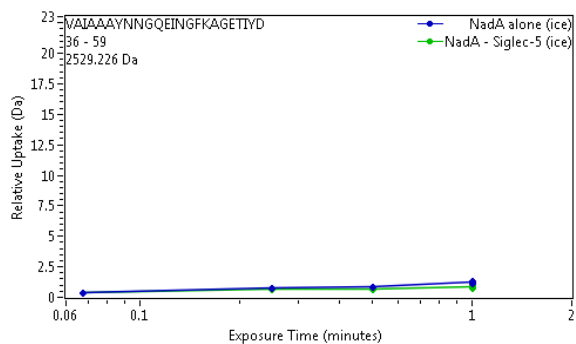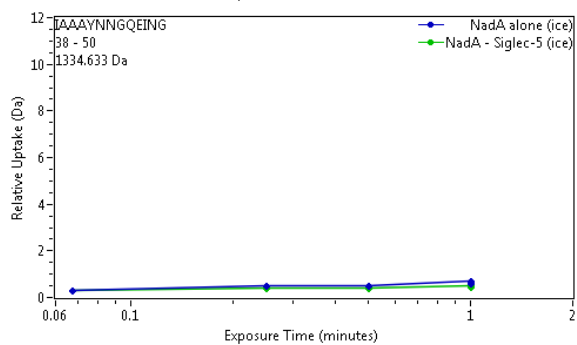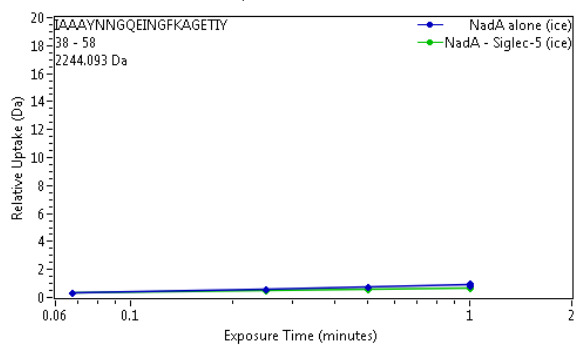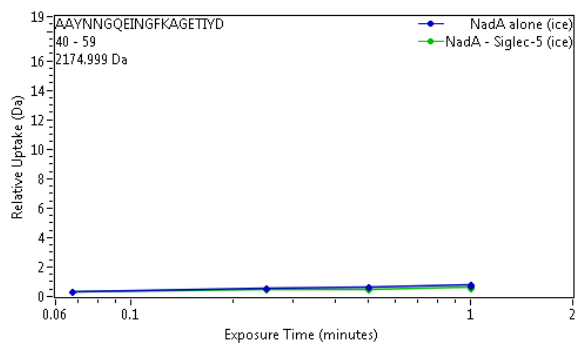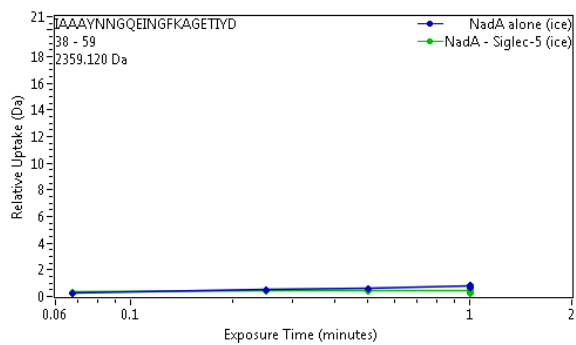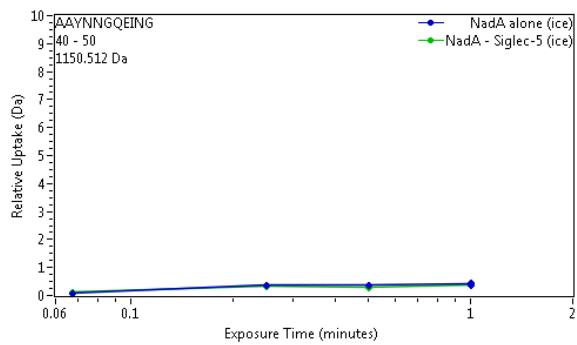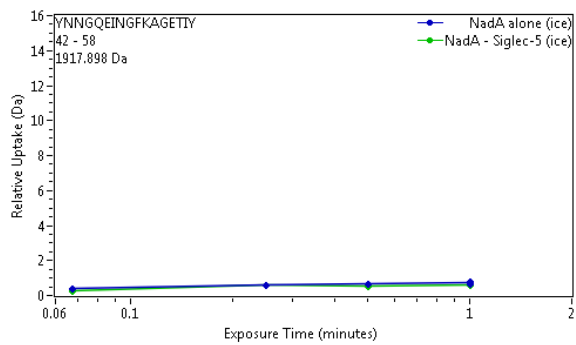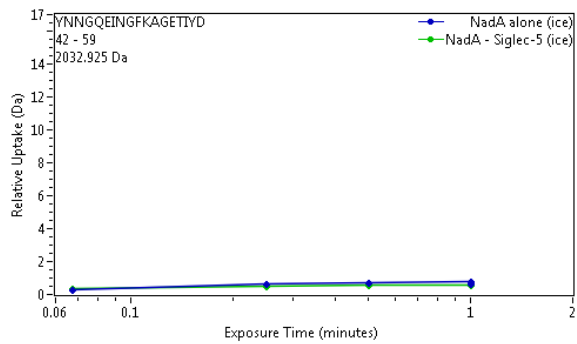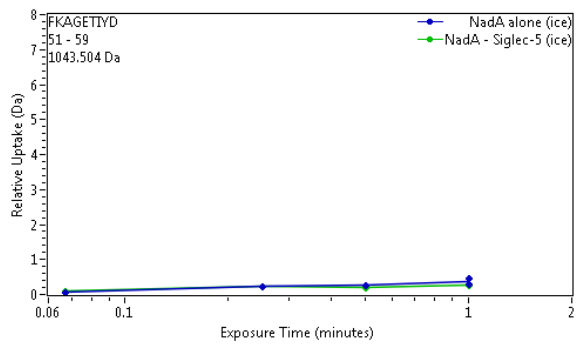

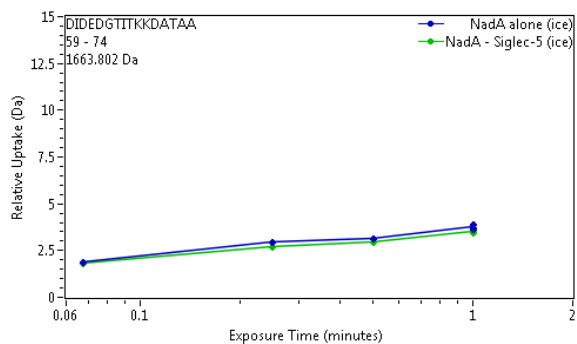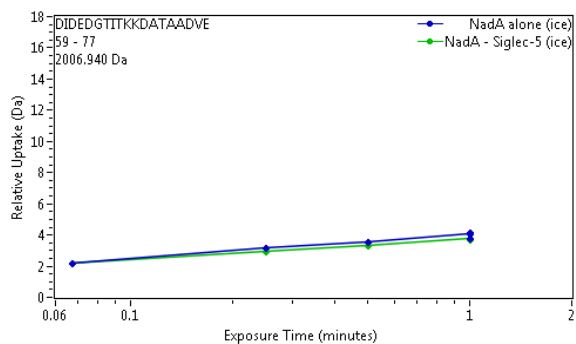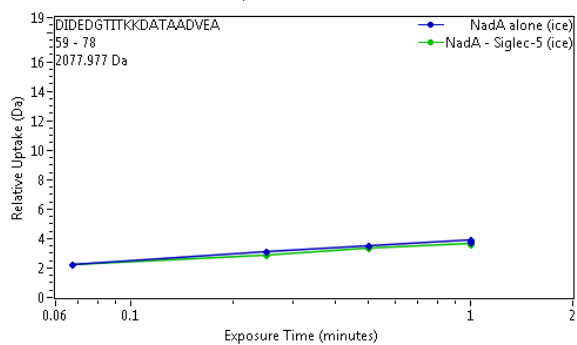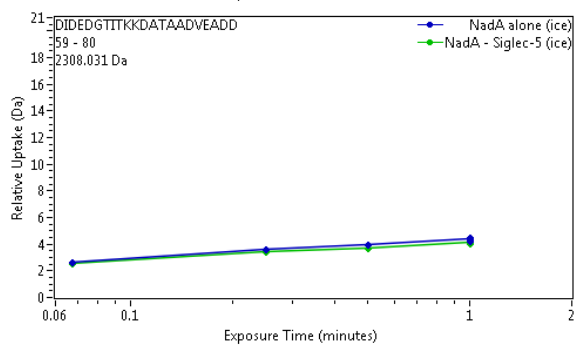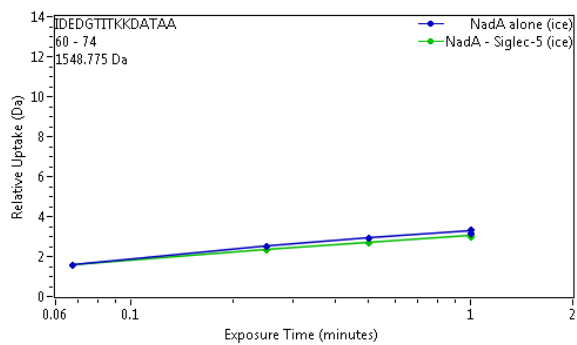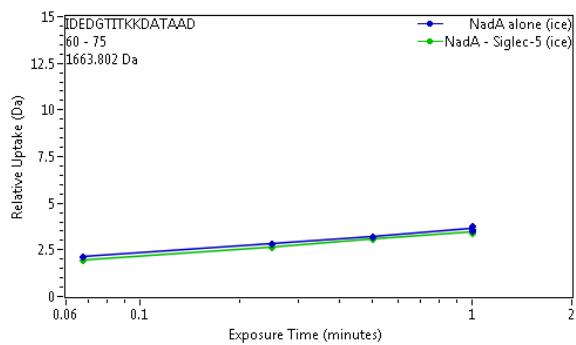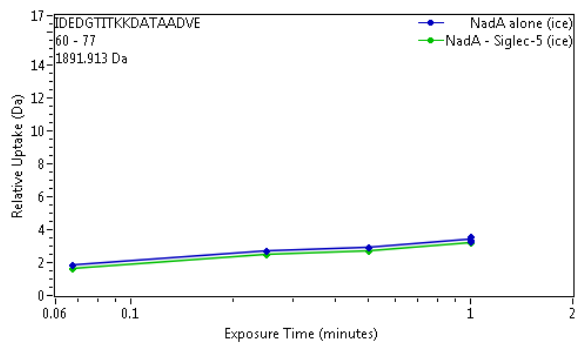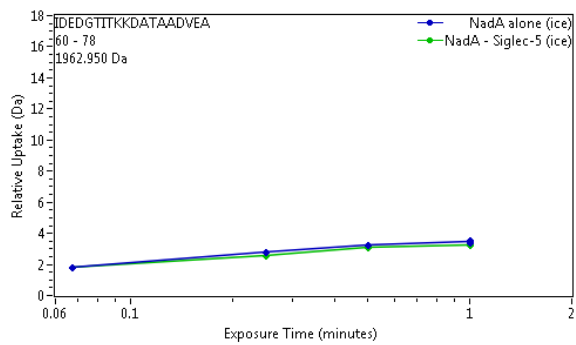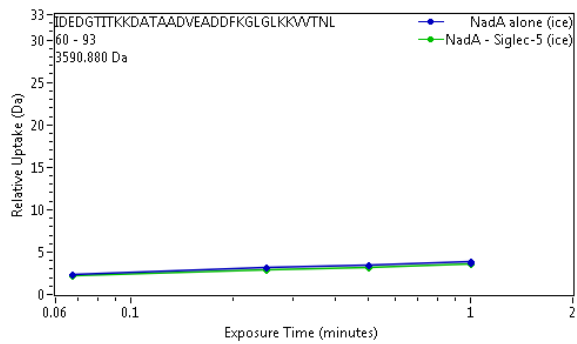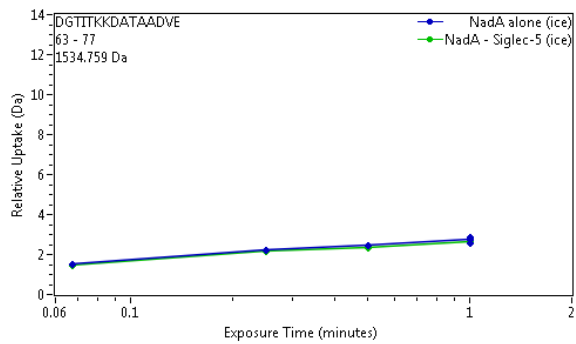

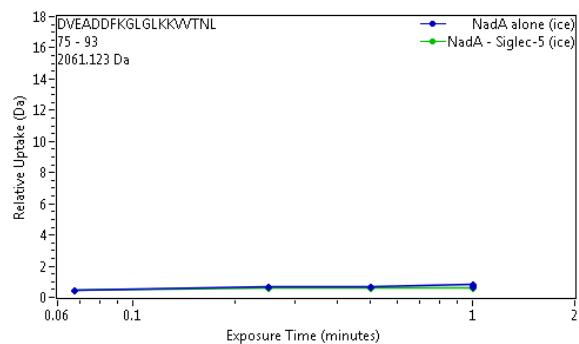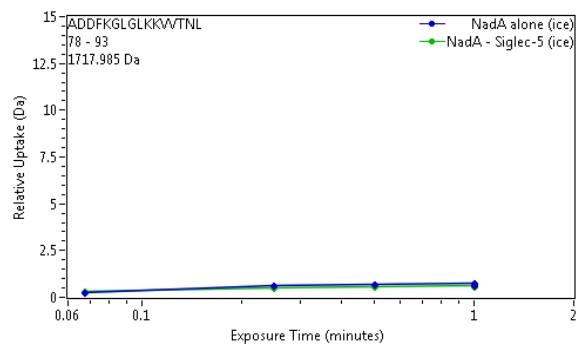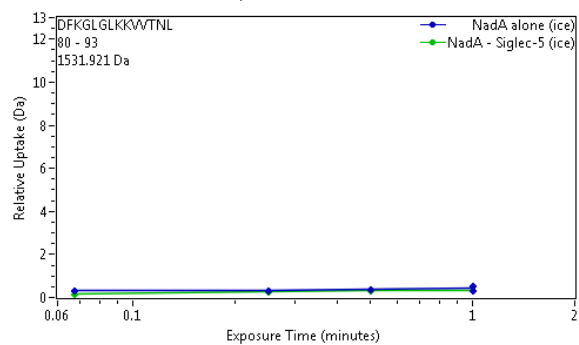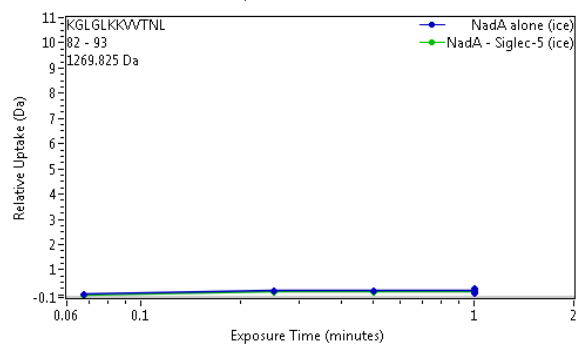

Supplement: Fig S3 — Summary of HDX effects in NadA head domain. [file mbio.01107-24-s0003.pdf]
