# Supplementary material for: Neisserial adhesin A (NadA) binds human Siglec-5 and Siglec-14 with high affinity and promotes bacterial adhesion/invasion
Source: mBio. 2024 Jul 23;15(8):e01107-24. doi: 10.1128/mbio.01107-24 (PMC11323535; doi:10.1128/mbio.01107-24)
Supplement: Legends — to supplemental material. [file mbio.01107-24-s0004.docx]

**Legends to Supplemental Material**

**Table S1.** Complete list of soluble recombinant human and mouse species spotted on microarray.

**Table S2.** HDX summary table.

**Table S2.** Peptide level differences in deuterium uptake.

**Fig. S1**. Deuterium uptake plots of peptides spanning residues 24-93 as exported from DynamX 3.0, showing the relative deuterium content upon labelling at RT. On the x-axis, the exposure time to the deuterated buffer (minutes); on the y-axis, the deuterium uptake Da).

**Fig. S2**. Deuterium uptake plots of peptides spanning residues 24-93 as exported from DynamX 3.0, showing the relative deuterium content upon labelling on ice. On the x-axis, the exposure time to the deuterated buffer (minutes); on the y-axis, the deuterium uptake (Da).

**Fig S3**. Peptides spanning residues 24-93 are depicted along the protein sequence and structural motifs of NadA head, wings and initial part of the stalk (PDB: 6EUN, sequence view). Peptides are coloured in blue if displaying a difference in HDX when NadA is incubated with Siglec-5 on ice or at room temperature, or in brown if no difference in deuterium content is observed in any HDX experiment and at any time point. Peptide are listed on the left, according to their position from the N- to the C-terminus. Dotted lines indicate the N-terminal amide of each peptide, whose HDX is not described within the segment considered, as always back-exchanged. NadA residues conformationally impacted by Siglec-5 are colored in blue.
